# Proteomic Analysis of Estrogen-Mediated Enhancement of Mesenchymal Stem Cell-Induced Angiogenesis In Vivo

**DOI:** 10.3390/cells10092181

**Published:** 2021-08-24

**Authors:** Maria Cristina Mihai, Mirel Adrian Popa, Viorel Iulian Șuică, Felicia Antohe, Edwin K. Jackson, Brigitte Leeners, Maya Simionescu, Raghvendra K. Dubey

**Affiliations:** 1 Institute of Cellular Biology and Pathology “Nicolae Simionescu” of the Romanian Academy, 050568 Bucharest, Romania; cristina.corotchi@gmail.com (M.C.M.); mirel.popa@icbp.ro (M.A.P.); viorel.suica@icbp.ro (V.I.Ș.); felicia.antohe@icbp.ro (F.A.); maya.simionescu@icbp.ro (M.S.); 2 Department of Pharmacology and Chemical Biology, University of Pittsburgh, Pittsburgh, PA 15219, USA; edj@pitt.edu; 3 Department for Reproductive Endocrinology, University Zurich, CH 8952 Schlieren, Switzerland; Brigitte.leeners@usz.ch

**Keywords:** adult stem cells, heart repair, tissue regeneration, women’s health, cardiovascular disease, capillary, sex hormones

## Abstract

Therapeutic use of mesenchymal stem cells (MSCs) for tissue repair has great potential. MSCs from multiple sources, including those derived from human umbilical matrix, namely Wharton’s jelly, may serve as a resource for obtaining MSCs. However, low in vivo engraftment efficacy of MSCs remains a challenging limitation. To improve clinical outcomes using MSCs, an in-depth understanding of the mechanisms and factors involved in successful engraftment is required. We recently demonstrated that 17β-estradiol (E2) improves MSCs in vitro proliferation, directed migration and engraftment in murine heart slices. Here, using a proteomics approach, we investigated the angiogenic potential of MSCs in vivo and the modulatory actions of E2 on mechanisms involved in tissue repair. Specifically, using a Matrigel^®^ plug assay, we evaluated the effects of E2 on MSCs-induced angiogenesis in ovariectomized (OVX) mice. Moreover, using proteomics we investigated the potential pro-repair processes, pathways, and co-mechanisms possibly modified by the treatment of MSCs with E2. Using RT-qPCR, we evaluated mRNA expression of pro-angiogenic molecules, including endoglin, Tie-2, ANG, and VEGF. Hemoglobin levels, a marker for blood vessel formation, were increased in plugs treated with E2 + MSCs, suggesting increased capillary formation. This conclusion was confirmed by the histological analysis of capillary numbers in the Matrigel^®^ plugs treated with E2 + MSC. The LC-MS screening of proteins obtained from the excised Matrigel^®^ plugs revealed 71 proteins that were significantly altered following E2 exposure, 57 up-regulated proteins and 14 down-regulated proteins. A major result was the association of over 100 microRNA molecules (miRNAs) involved in cellular communication, vesicle transport, and metabolic and energy processes, and the high percentage of approximately 25% of genes involved in unknown biological processes. Together, these data provide evidence for increased angiogenesis by MSCs treated with the sex hormone E2. In conclusion, E2 treatment may increase the engraftment and repair potential of MSCs into tissue, and may promote MSC-induced angiogenesis after tissue injury.

## 1. Introduction

Cardiovascular disease is the leading cause of mortality and morbidity in women [1]. Although multiple therapeutic approaches, including changes in life style, drugs, and surgery, are available to treat/manage various heart conditions, heart transplantation remains the only option for patients with severely advanced heart disease. As the availability of donor hearts is limited, alternative approaches, including tissue repair or regeneration using growth factors and stem cells, are being intensively evaluated.

Use of stem cells to repair damaged hearts has been investigated for over two decades. Ethical concerns with use of embryonic stem cells have led to the identification of alternative adult resources, including mesenchymal stem cells (MSCs) [2]. Use of bone marrow derived MSCs for tissue repair/regeneration has been extensively studied and considered a gold standard for multiple clinical applications of MSCs [3]. However, based on basic and clinical findings over the last decade, it has become increasingly clear that bone marrow may not be the most appropriate source for the collection of MSCs. Apart from the invasive and painful isolation procedure, the cell density of MSCs in bone aspirates is markedly low (approximately 0.001–0.01%) [3,4]. Moreover, their qualitative and quantitative properties decrease with the donor’s age [4]. In addition to bone marrow, there are multiple other sources for MSCs, including adipose tissue, placenta, gut, lung, liver, amniotic fluid, dental pulp, periodontal pulp, and heart tissue [5]. MSCs derived from Wharton’s Jelly (WJ) are considered to be a valuable alternative source of stem cells that possess multipotent properties that lie between embryonic and adult stem cells [6,7]. Indeed, WJ-MSCs, also called umbilical matrix-derived cells, have exceptional properties and are bonafide MSCs [8].

MSCs are of mesodermal origin and are capable of differentiating into various cell types (endothelial cells, osteoblasts, chondrocytes, adipocytes, and myocytes) when challenged with specific growth factors, signaling molecules, or transcription factors [5]. In practical terms, MSCs can be grown in vitro and primed for delivery [5]. The multiple tissue sources and pluripotent nature of MSCs, combined with the ease of in vitro expansion, makes MSCs an attractive therapeutic tool for treating heart damage. Indeed, encouraging progress has been made in the development of pluripotent adult stem/precursor cell approaches as a therapeutic tool to cure/repair the damaged heart following myocardial infarction or heart failure [4,5,9]. Results from many (but not all) animal and clinical trials have demonstrated neovascularization, scar reduction, and functional recovery using MSCs.

Although attractive, a major limitation of MSCs therapy is the low engraftment yield of transplanted cells in infarcted zones; approximately only 0.44% of transplanted MSCs reside in the myocardium after 4 days [3,10]. Moreover, direct intra-cardiac delivery of MSCs does not substantively increase their engraftment yield (range 1–3%) [11]. Clearly, a better understanding of MSCs biology is required to optimize MSCs therapy [12]. Moreover, identifying molecules that increase homing and retention of MSCs in target tissues would be a major step forward [12]. Hence, there is a major need for investigating therapies which may be effective and beneficial against advanced heart disease [9], and which may provide short- or long-term relief/protective actions in patients requiring heart transplantation [4,5].

Proteins play a prominent role in regulating numerous biological and physiological processes in cells, tissues, and organs. Proteins have unique functions and play a crucial role in regulating tissue/organ growth, development, and metabolic regulation, as well as disease progression and pathophysiology. Moreover, proteins act as signatures and are extremely useful in defining/characterizing the effects of treatments on cells and tissues, including processes such as tissue regeneration.

Proteomics is the study of the suite of proteins that are expressed, including their various isoforms; thus, proteomics can clarify the different biological mechanisms that control the behavior of cells and organisms [13]. Data obtained from a proteomic analysis can reveal numerous biological and pathophysiological information regarding drug–cell interactions and effects of treatments [13]. The identified proteins that are significantly expressed can serve as therapeutic and diagnostic markers for tissue regeneration. By using advanced proteomics technologies, it is possible to identify the differentially expressed proteins (DEPs) and determine their functions, interactions, and structural changes following treatment.

An important issue is the impact of sex hormones in MSCs therapy [14]. Previously, we demonstrated that 17β-estradiol (E2), via both subtypes of the estrogen receptor, can stimulate proangiogenic responses in WJ-MSCs, in vitro [15]. In this regard, using in vitro and ex vivo studies, we showed that priming of MSCs with E2 induces MSCs proliferation and migration and boosts their ex vivo incorporation towards/into tissue cardiac slices [15]. Moreover, using specific gene and protein detection methods, including mass-spectrometry assays, we revealed the up-regulation and enhancement of proangiogenic molecules in MSCs following treatment with E2. Our observations are supported by the facts that: (1) E2 promotes angiogenesis by stimulating progenitor endothelial and stem cells [16,17,18]; (2) MSCs acutely treated with E2 improve myocardial recovery after ischemia [19]; (3) female MSCs generate more myocardial protective paracrine factors than do male MSCs [20]; (4) the protective effects of E2 on cardiovascular health of women is well established [21]; and (5) within the reproductive system, local (follicular) increases in E2 trigger tissue remodeling processes, e.g., ovulation [22]. However, whether in vivo E2 promotes proangiogenic and tissue regenerative processes by WJ-MSCs, and the mechanism’s involved, remains unknown. If E2 can facilitate the repair process in a damaged heart, E2 may help tissue repair directly or by improving stem cell function in a gender specific fashion.

In the present study, we extended our experiments to assess the impact of E2 on MSCs-induced angiogenesis in vivo. Here, we implanted in OVX mice Matrigel^®^ plugs containing WJ-MSCs primed with or without E2. We then assessed angiogenesis and conducted an in vivo proteomic analysis to reveal/screen the pro-regenerative processes and biological pathways that may contribute to or promote E2-induced integration of MSCs into cardiac tissue. We report that E2 enhances capillary formation by MSCs in vivo and that these effects are accompanied by upregulation of key pro-angiogenic and tissue regenerative pathways.

## 2. Materials and Methods

All information regarding Animals and major resources used is provided in Table S1.

### 2.1. Reagents for Proteomics

Mass spectrometry or electrophoresis grade reagents were employed for LC-MS studies. Ethylenediaminetetraacetic acid (EDTA), acetone, formic acid, acetonitrile, sodium deoxycholate (DOC), ammonium bicarbonate, DL-dithiothreitol (DTT), iodoacetamide (IAA), Trizma^®^ hydrochloride (Tris-HCl), n-acetyl-l-cysteine (NAC), urea, and water were procured from Sigma-Aldrich (St. Louis, MO, USA). Trypsin (Gold grade) was from Promega (Madison, WI, USA), whereas protease inhibitor Complete cocktail was from Roche (Basel, Switzerland). C18 columns for solid phase extraction were from Waters (Milford, MA, USA). Advanced Protein Assay ADV-01A (Tebu-Bio, Cytoskeleton, Denver, CO, USA) kit was used for protein quantification.

### 2.2. Cell Culture-Isolation and Characterization of WJ-Derived Mesenchymal Stem Cells

WJ-MSCs that were previously isolated and characterized by Corotchi et al. [7,15], were used in this study. For the original preparation, human umbilical cord samples were obtained following the guidelines required by EU (European Union) and national legislation regulation with respect to human samples collection and personal data collection and protection. Ethical Commission of Institute of Cellular Biology and Pathology “Nicolae Simionescu” approved all protocols and experiments in conformity with the edited Declaration of Helsinki related to human subjects [7].

Wharton’s jelly (WJ) was isolated from the human umbilical cord samples after detaching umbilical cord vein and arteries and removing amniotic epithelium, as we previously described [15]. Subsequently, MSCs were isolated following enzymatic digestion of the connective tissue with collagenase I and hyaluronidase (Sigma-Aldrich, St. Louis, MO, USA) and were characterized as previously described [7].

The MSCs used express both estrogen receptors α and β and was confirmed by us previously using Western blotting [15] and shown in Figure S1A.

### 2.3. Proliferation of MSCs

WJ-derived MSCs were seeded onto 75 cm^2^ flasks (25 × 10^4^ cells) and maintained in culture for 4 days, in presence or absence of 100 nM of E2. The morphology of E2-stimulated WJ-MSCs was confirmed microscopically using light/phase-contrast and fluorescent microscopy and a digital camera system for imaging (Eclipse TE300 and Digital Net Camera DN100, Nikon, Tokyo, Japan). For cell viability and proliferation, we used the techniques as previously described [7].

### 2.4. Angiogenesis In Vivo Studies

The process of angiogenesis in vivo was investigated using the Matrigel^®^ plug technique. Mus musculus species (female C57Bl/6) were purchased from The Jackson Laboratory (Bar Harbor, ME, USA). C57Bl/6 mice were bred and maintained in SPF conditions at the animal facilities of the Institute of Cellular Biology and Pathology “Nicolae Simionescu”. We performed all the in vivo experiments in compliance with the “Guide for Care and Use of Laboratory Animals” published by National Institutes of Health US (NIH 85-23, review of 1996) and approved by the Ethical Committee of the Institute of Cellular Biology and Pathology “Nicolae Simionescu”. All the animals used in our in vivo experiments received a typical diet including free access to food and water. Female C57B1/6 mice 8–9 weeks age were divided into 2 groups: control (intact; *n* = 6) and another subjected to ovariectomy (OVX) (*n* = 6). An additional group of 6 animals was used for the pilot experiment (human fibroblasts—NUFFs). Ten days before Matrigel^®^ plug implantation, mice were immunosuppressed with cyclosporine-A daily (2 mg/100 µL/mouse/day) via intraperitoneal injection.

The operating table was rendered germ-free using 70% ethanol, followed by a 30-min sterilization with a UV lamp. The surgical instruments were sanitized by autoclaving and sterilized for 5 s at 250 °C using GerminatorTM-500 apparatus (Braintree Scientific, Inc., Braintree, MA, USA). Animals were anesthetized by i.p. injection using a mix of ketamine and xylazine (ratio 1/10). After anesthesia, C57Bl/6 females were shaved in the dorsal region and cleansed with 70% ethanol before surgery. Throughout surgery, animals were maintained on a rectangular continuous heating device and the body temperature was continuously monitored and controlled at 37 ± 0.5 °C. To relieve post-operative pain, we used Rimadyl and eye protection gel. After removal of the ovaries, C57Bl/6 females recovered from anesthesia in ~2 h. Intact or “sham-operated” control female mice were subjected to the same surgical treatment, except oophorectomy. The animals were monitored daily for three weeks.

Enzyme-Linked Immunosorbent Assay (ELISA; absorbance at 450 nm) was utilized to detect E2 concentrations in the bloodstream and to confirm successful oophorectomy. In the 96-well pre-coated anti-E2-IgG plate, we added the samples of interest and the conjugate E2-HRP, where E2 from the samples of interest competes with the E2-HRP conjugate for antibody binding. After incubation, all wells were washed to remove unbound material. Subsequently, we added the TMB-HRP substrate that produced a blue color. The reaction was quenched after adding the Stop solution (according to manufacturer’s instructions). The intensity of the signal was inversely proportional to the amount of E2 in our samples. Absorbance was measured at a wavelength of 450 nm.

### 2.5. Matrigel^®^ Plug In Vivo Implantation

The Matrigel^®^ reagent was thawed at 4 °C before use. On ice, we prepared a mix containing 450 µL of Corning Matrigel^®^ Growth Factor Reduced (GFR) Basement Membrane Matrix (Corning Inc., New York, NY, USA) and 50 µL of cell suspension (MSCs) ± E2. The mixture was inserted into 11- to 12-week-old C57Bl/6 females (INTACT or OVX animals) under ketamine/xylazine anesthesia given by i.p. injection. The Matrigel^®^ reagent and other consumables were preserved on ice until injection to prevent solidification. Using 25-gauge needles, we inserted the Matrigel^®^ mix ± MSCs ± E2 to each female mouse: 2 insertions in the dorsal surface and one in the paraspinal space. On day 7, Matrigel^®^ implants were excised and used for further analysis.

### 2.6. Hemoglobin Assay by Drabkin Method

Initially, Matrigel^®^ implants are avascular and possess a transparent color. We employed Drabkin method to quantitatively measure hemoglobin within the implant as an index of angiogenesis. Quantifying the hemoglobin content allowed detection of new vessels within the Matrigel^®^ plugs. The excised plugs were weighed and homogenized in a hypotonic lysis buffer containing Brij solution for 5–10 min on ice. Hemoglobin concentration is interpolated using a standard curve and the calculated values were expressed as g/dL hemoglobin/plug weight as previously described [23].

### 2.7. Microtome Sectioning and Histology of Matrigel^®^ Plugs

After 7 days, C57Bl/6 mice were sacrificed by carbon dioxide inhalation; the Matrigel^®^ plugs were excised and kept in 4% buffered paraformaldehyde for 24 h and thereafter fixed and embedded in paraffin. Matrigel^®^ implant sections were cut (4 µm thickness) using the DTK-2000 microtome (Dosaka, Kyoto, Japan). Sections were stained with hematoxylin/eosin and DAPI according to standard protocols.

### 2.8. Liquid Chromatography–Mass Spectrometry (LC–MS) Analysis of Matrigel^®^ Plugs

After excision of all plugs from OVX female mice and control animals, we proceeded to homogenize the Matrigel^®^ plugs for proteomic analysis of the resulting supernatants. For sample preparation of LC-MS analysis, we washed the cells with PBS 1X solution, centrifuged them, and solubilized the resulting pellet in denaturing buffer (8 M urea, 1% DOC, 0.1% Tris-HCl—pH 8.8) and protease inhibitors through sonication for 30 s on ice (UP50H Ultrasonic Processor, Teltow, Germany). The cellular debris was discarded following centrifugation at 18,000× *g* and the supernatant used for measurement of protein quantity using the Pierce BCA colorimetric technique (Thermo Scientific, Rockford, IL, USA). The samples were precipitated with acetone and the carbamidomethylated proteins subjected to the proteolysis process followed by purification using solid phase extraction. Materials utilized are listed under Major-resource table. We rigorously followed protocols as previously described [15]. The LC–MS analysis was performed using EASY n-LC II system (Thermo Fisher Scientific, Carlsbad, CA, USA) while mass spectra were obtained with a 60k-LTQ mass spectrometer—Orbitrap Velos Pro (Thermo Fisher Scientific, Carlsbad, CA, USA) [24].

Proteome Discoverer 1.4 (Thermo Scientific) managed the protein identification in UniProtKB/SwissProt *Homo sapiens* fasta database using Mascot 2.5.1 (Matrix Science, London, UK) search algorithm. The dynamic modifications comprised oxidation of methionine and deamidation of asparagine and glutamine while carbamidomethylation of cysteine was set as a static modification. The peptide target False Discovery Rate (FDR) was < 0.05. Sieve 2.1 (Thermo Fisher Scientific, Carlsbad, CA, USA) was employed for label-free relative quantification analysis, in which a spectral abundance alteration of at least 1.5-fold was considered significant if the *p*-value was < 0.05. Thereafter, we used FunRich (version 3.1.3) bioinformatics software to assess the functional enrichment analysis to identify overrepresented classes. Additionally, we performed analyses to attain the biological pathways, gene ontology categories, protein domains, site of expression, transcription factors, clinical phenotypes, extracellular vesicles, miRNA enrichment, protein interaction network, and cross database accession conversion. Moreover, we used FunRich to evaluate miRNAs enrichment analysis to identify biological pathways that may be perturbed. For more in-depth information, we used Reactome database tool to reveal the most important biological processes and pathways show the over-representation results that were obtained as a list of statistically over-represented pathways and sub-pathways.

The third analysis that we have employed for our proteomic data was using the Panther bioinformatics software or Protein Analysis Through Evolutionary Relationships. As part of the Gene Ontology Reference Genome Project, Panther allows us to further classify and identify our data by the functionality of the proteins and their genes for high-throughput analysis. Panther analysis allows us to discover the functionality processes of proteins/genes, such as pathways, biological processes, molecular functions, and cellular components. Processing and proteomic analysis of the data was performed in three biological replicates; each technological replicate was processed and analyzed for describing the proteomics roadmap.

### 2.9. Quantitative Real-Time PCR Analysis

Total RNA isolation was made by using PureLinkTM RNA Mini Kit (Life Technologies^TM^, Carlsbad, CA, USA) according to manufacturer’s instruction. The total cDNA volume utilized in RT-qPCR analysis was 10 µL per well in MicroAmp Fast Optical 384-well Reaction plates (Applied Biosystems, Beverly, MA, USA). The 384-well plates were sealed using the MicroAmp Optical Adhesive Film (Applied Biosystems, Beverly, MA, USA) and for a short time pulsed to 300 g prior to the RT-qPCR analysis. The endogenous control used was GAPDH for target gene mRNAs.

The specific oligonucleotide primers employed to determine the quantitative mRNA expression for Ang-1(angiopoietin 1), Tie-2 (angiopoietin receptor 2), VEGF-A, vascular endothelial growth factor receptor 2 (VEGFR-2), and Endoglin are shown in Table S2. Quantitative real-time PCR data were analyzed by SDS2.4 Standalone software (Applied Biosystems, Beverly, MA, USA). Notable *p* values were determined from comparative Ct (ΔΔCt) to determine the relative gene expression.

### 2.10. Statistical Analysis

All studies were performed at least in triplicate. Quantifications are expressed as the mean ± SEM. For comparison between two groups, the 2-tailed Student’s *t*-test was utilized. A difference between experimental groups was considered significant when the *p*- value was less than 0.05.

## 3. Results

We have previously shown the pro-angiogenic and tissue regeneration potential of E2-treated MSCs in vitro; hence, the current study was focused on the in vivo analysis of E2-stimulated MSCs secretome by using Nano Liquid Chromatography—Mass Spectrometry analyses. We evaluated the pro-angiogenic, or tissue regeneration, potential induced by E2 on MSCs using the in vivo Matrigel^®^ plug assay. Intact or OVX C57Bl/6 females were subcutaneously injected with Matrigel^®^ only or with or without E2 and MSCs.

**Figure 1:**
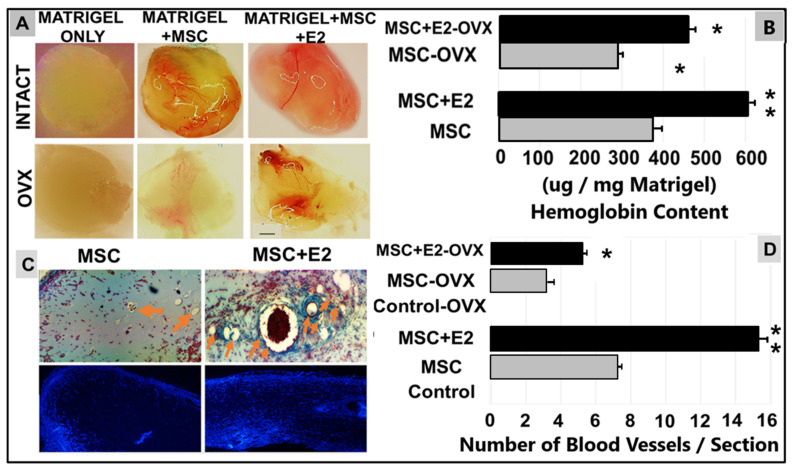
E2 enhances angiogenesis effects in Matrigel^®^ plugs containing MSCs. (**A**) Exhibits a macroscopic view of Matrigel^®^ plugs from each group—sham-operated or OVX—in presence or absence of E2 (100 nM): C57Bl mice injected with Matrigel^®^ only, Matrigel^®^ + cells (Matrigel^®^ + MSC), or Matrigel^®^ + hormone-treated cells (Matrigel^®^ + MSC + E2). (**B**) Displays measures of hemoglobin content using Drabkin’s reagent kit of Matrigel^®^ implants from both groups. The levels of this angiogenesis marker were amplified in MSCs stimulated with the sex hormone (MSC + E2 ± OVX) in comparison with control (MSCs only). Each value corresponds to mean ± SEM (*n* = 3); * *p* < 0.05, ** *p* < 0.01 compared with control. (**C**) Descriptive photomicrographs of Matrigel^®^-derived blood capillaries/vessels embedded in paraffin and stained with hematoxylin & eosin, and DAPI; Orange arrows indicate blood vessel structures; scale bar—100 μm. (**D**) Quantification of the new blood vessels was presented as mean ± SD (*n* = 2), ** *p* < 0.01, and * *p* < 0.001 compared with control. Note: E2, 17-beta estradiol; MSCs, Wharton’s jelly-derived mesenchymal stem cells; OVX, ovariectomy surgery.

After 7 days, Matrigel^®^ implants were removed from the animals and subject to further analysis. Measurements of the pro-angiogenic responses in the Matrigel^®^ plug assay were achieved by quantification of the hemoglobin content of the implants or by histological analysis of Matrigel^®^ plug sections. As shown in Figure 1A, Matrigel^®^ implants containing E2 and MSCs (E2 + MSCs) induced vascular development and a clear angiogenic response (highlighted by the red capillaries that extended within the whole plug) contrary to what was detected in the Control where no E2 or MSCs were embedded (Matrigel^®^ only). The hemoglobin (Hb) levels were significantly increased in the Matrigel^®^ implants with E2 + MSCs in comparison with the Control (Figure 1B).

Prior treatment of MSCs with E2 revealed the amplification of pro-angiogenic effects in the Matrigel^®^ plugs in contrast to Matrigel^®^ only. This neo-vessel formation in the implants was verified macroscopically and through histology with H&E staining (Figure 1C). The presented in vivo experiments support our previous in vitro angiogenesis and tissue regeneration-related studies and confirm that, after one week, implanted E2-MSCs in Matrigel^®^ plugs in vivo were able to induce vessel formation de novo in comparison with Control and to amplify angiogenesis in the Matrigel^®^ plugs containing treated cells.

### 3.1. Nano Liquid Chromatography-Mass Spectrometry (LC-MS) Analysis of the Matrigel^®^ Implants

After surgically removing the Matrigel^®^ plugs, we proceeded to enzymatic digestion and proteomic analysis. The injected peptide quantity was normalized for each sample by absorbance readings at 280 nm using the Pherastar FS system and the LVis attachment (BMG Labtech). For peptide separation and identification, we employed the Easy nLC II nano-chromatographic system coupled to the LTQ Orbitrap Velos Pro hybrid mass spectrometer (Thermo Fisher Scientific, Carlsbad, CA, USA).

**Figure 2:**
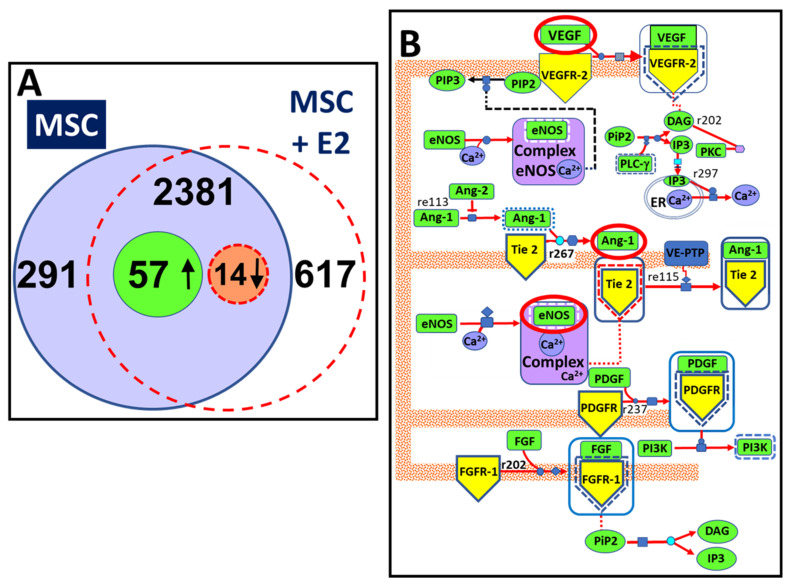
Liquid chromatography-mass spectrometry (LC-MS) testing revealed that E2 up-regulated proteins involved in angiogenesis and tissue remodeling. (**A**) represents the entire proteomic profile data of the in vivo Matrigel^®^ plugs for each group (sham-operated and OVX) after LC-MS and bioinformatic analysis. A total of 2381 proteins were collectively identified in both control implants and Matrigel^®^ plugs containing MSCs treated with E2. The relative quantitative analysis revealed 57 up-regulated and 14 down-regulated proteins (*p*-value < 0.05). (**B**) illustrates statistical analysis (using Panther) of the protein pathways modulated by E2 presence in MSCs. Analyzing the proteins from excised Matrigel^®^ plugs (containing E2 treated MSCs), we discovered proteins mainly involved in angiogenesis processes. VEGF (vascular endothelial growth factor), Ang-1 (angiopoietin 1), Tie-2 (angiopoietin receptor 2), and eNOS (endothelial nitric oxide synthase).

The bioinformatic and statistical analysis of the data obtained using the nano LC-MS revealed that E2 up-regulated proteins involved in angiogenesis and tissue remodeling. As seen in Figure 2A, the total proteomic profile analysis of the in vivo Matrigel^®^ plugs for each group (sham operated and OVX) revealed 2381 proteins identified in both control plugs and plugs treated with MSCs + E2. Relative quantitative analysis revealed 57 up-regulated and 14 down-regulated proteins (*p*-value < 0.05) due to E2 treatment of the MSCs.

**Figure 3:**
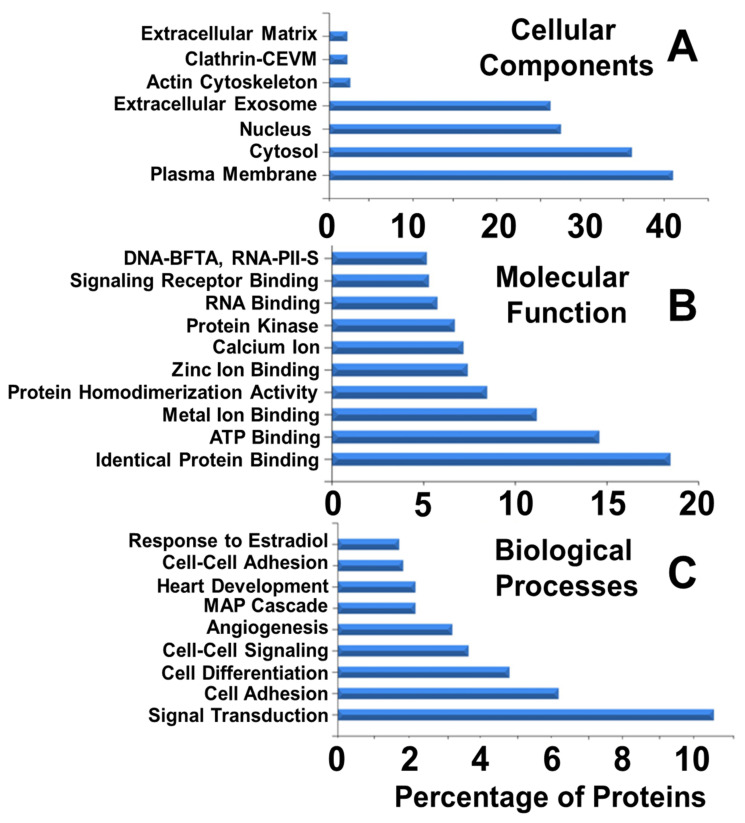
Analysis of the differentially expressed proteins modulated by E2 shows a significant over-representation (False Discovery Rate corrected *p*-value < 0.05) of the proteins participating in biological pathways, biological processes, cellular components and molecular functions. FunRich analysis of all the 2381 proteins which were commonly identified in both vehicle treated control MSCs and MSCs treated with E2 were characterized on their cellular components (**A**), biological pathways (**B**), molecular functions (**B**) and biological processes (**C**). DNA-BFTA, DNA-binding transcription factor activity; RNA-PII-S, RNA polymerase II-specific; and Clathrin-CEVM, Clathrin-coated endocytic vesicle membrane.

The bioinformatic analysis of the proteins modified by E2 stimulation allowed us to characterize them and reveal their over-representation in several Gene Ontology categories and interaction-based networks such as cellular component by E2 (Figure 2B). The proteomic data is deposited at the ProteomeXchange consortium via PRIDE partner repository with dataset identifier PXD026380 and 10.6019/PXD026380. As shown in Figure 2A, using computer-based analysis we identified 3289 proteins in MSCs using a MudPIT-like (multidimensional protein identification technology) approach, where spectral data from all the replicates were combined. Out of the 3289 proteins, 2381 proteins were commonly identified in both vehicle treated control MSCs and MSCs treated with E2 implanted for 7 days (Figure 2A). Using chromatographic alignment and precursor ion-intensity comparison, a relative quantitative profile of the E2 treatment was obtained, as previously described [15]. Importantly, 291 proteins were uniquely identified in control cells, whereas 617 proteins were uniquely identified in E2 treated MSCs. Out of the commonly identified proteins, 71 were significantly altered by E2 treatment, with 57 proteins upregulated (ratio of MSC + E2/MSC ≥ 2.12) and 14 proteins down-regulated (ratio of MSC + E2/MSC ≤ 0.47). Among the proteins upregulated were those associated with angiogenesis and tissue remodeling (signal transduction, cell–cell adhesion and signaling, cell differentiation and adhesion, extracellular exosomes and matrix, cytosolic and plasma membrane proteins, ATP binding, MAPK cascade, and protein kinase binding; Figure 2B and Figure 3). A map of detected Uniprot IDs statistically analyzed by Reactome software revealed significant (*p* < 0.01 vs. MSC alone) upregulation of proteins involved in specific cellular homeostasis (Figure S2).

Using FunRich analysis (software version 3.1.3) we identified the proteins and pathways representing cellular components, molecular function and biological process modulated by E2 in MSCs (Figure 3A–C, respectively). The list of over-represented components/processes in response to E2 in implanted MSCs treated with E2 is provided in Supplementary Data. Over-represented proteins for cellular component, molecular function, and biological process are listed in Tables S3–S5, respectively. Moreover, the top ten proteins that were down and up regulated are shown in Table 1 and Table 2, respectively. We observed significant changes in protein class such as extracellular exosomes, cytosol, plasma membrane, Clathrin-coated endocytic vesicle membrane, extracellular matrix, DNA-binding transcription factor activity, metal ion and ATP binding, and identical protein binding (Figure 3A,B). Importantly, biological process analysis revealed a significant and noticeable upregulation of signal transduction, cell adhesion, cell differentiation, cell–cell signaling, and angiogenesis, as well as heart development (Figure 3C). Other pathways of relevance to tissue repair and remodeling that were upregulated were MAPK cascade, protein kinase binding, calcium binding, protein homo-dimerization activity, and signaling receptor binding.

**Figure 4:**
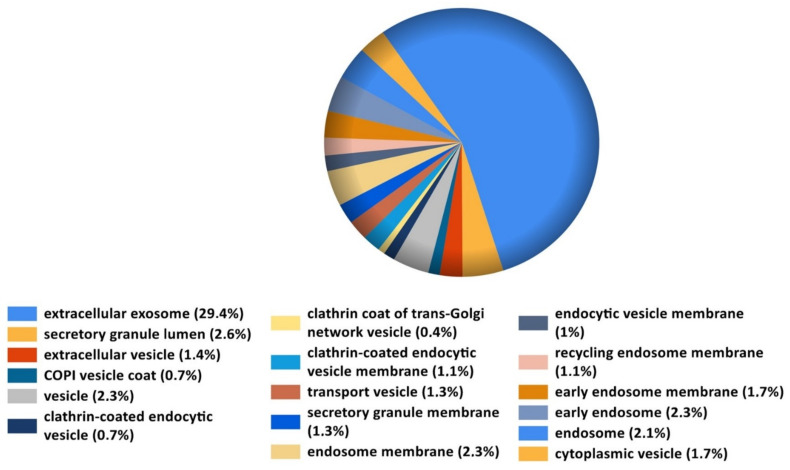
FunRich (Uniprot database) analyze of the proteins differentially expressed by E2 stimulation shows a significant over-representation (False Discovery Rate corrected *p*-value < 0.05) of the proteins participating in different cellular components and identified by Reactome pathways. The screening of the proteins involved in the paracrine function of the MSCs are represented as percentage (%) from the total of the cellular protein components.

Utilizing FunRich (Uniprot database) analysis of the proteins differentially expressed by E2 stimulation showed a significant over-representation (False Discovery Rate corrected *p*-value < 0.05) of proteins participating in different cellular components and identified by Reactome pathways. Figure 4 depicts E2 induced changes in MSCs proteins involved in the paracrine function. These proteins represented > 30% of all cellular component proteins, and largely represented extracellular exosomes (29.4%), vesicles, vesicle membranes, secretory granules, endosomes, and Clathrin-coated endocytic vesicles. As MSCs derived vesicles actively participate in tissue repair, their upregulation in MSCs by E2 may positively influence and enhance MSCs induced tissue repair. Analysis by Reactome pathways also revealed different percentages (%) of hormone associated cellular mechanisms in E2 treated MSCs (Figure S1B).

### 3.2. FunRich In Vivo Data Analysis of Proteins, Pathways, Site of Expression, and miRNA Enrichment Regulated by E2 in MSCs

**Figure 5:**
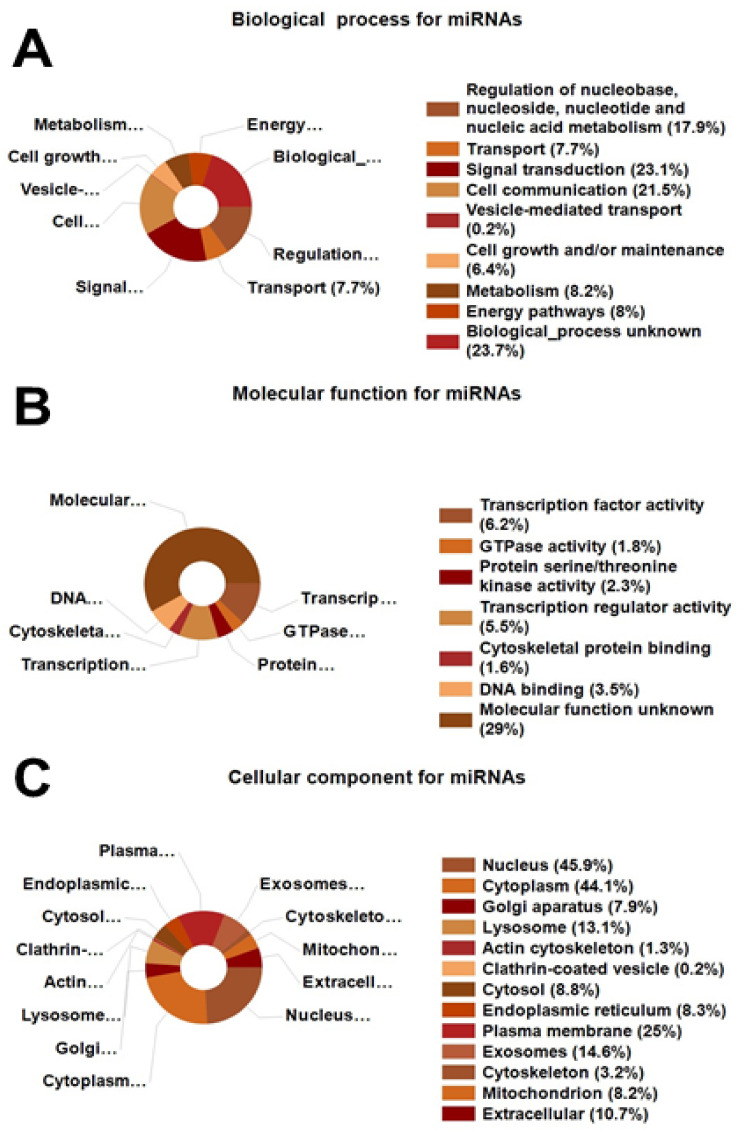
UniProt ID of the detected proteins reveals a total of 321 miRNAs through conversion by using FunRich miRNA enrichment tools. (**A**) shows miRNA enrichment based on UniProt ID protein’s conversion characterized on their biological process, (**B**) their molecular function, and (**C**) their cellular component.

Through conversion of UniProt ID of the detected proteins using FunRich miRNA enrichment tools, we uncovered a total of 321 miRNAs. The most important 100 miRNAs were characterized by their biological process, molecular function, and cellular component (Figure 5). These miRNAs were mainly involved in cellular communication, vesicle transport, and metabolic and energy processes; however, approximately 25% participated in unknown biological and molecular processes.

**Figure 6:**
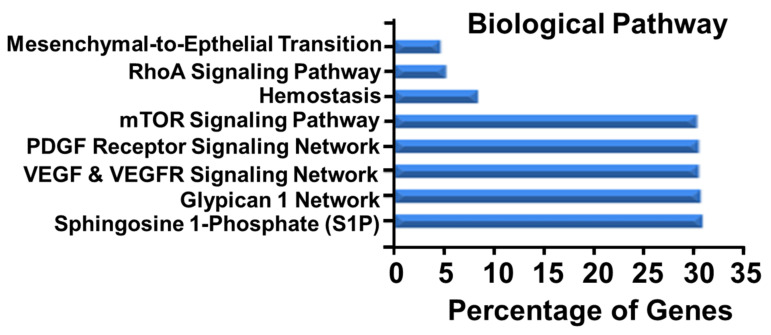
UniProt ID of the detected proteins reveal total of 321 miRNAs through conversion by using FunRich miRNA enrichment tools. Bar graph depicts miRNA enrichment based on UniProt ID protein’s conversion characterized on their Biological Pathway.

Statistical bioinformatic analysis of the labeled proteins (71 proteins) modified by E2 presence were characterized on their biological processes (Figure 5A), molecular function (Figure 5B) and cellular components (Figure 5C). Importantly, treatment with E2 upregulated genes within biological pathways that are relevant for tissue repair and remodeling (Figure 6). In this context, significant upregulation in mesenchymal to epithelial transition, RhoA signaling, mTOR signaling, PDGF receptor signaling, VEGF and VEGFR signaling and network, glypican 1, and sphingosine 1-phosphate were observed. Furthermore, site of expression (Figure S3) shows a significant over-representation (False Discovery Rate corrected *p*-value < 0.05) of all the analyzed data.

### 3.3. qRT-PCR Analysis of Matrigel^®^ Implants Reveals the Over-Expression of Pro-Angiogenic Genes by E2

**Figure 7:**
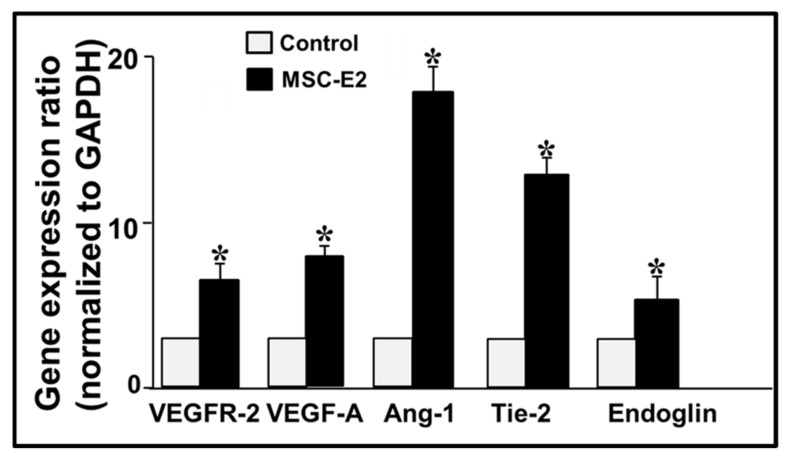
Quantification of pro-angiogenic genes in Matrigel^®^ plugs by RT-qPCR analysis shows enhanced levels of VEGFR-2, VEGF-A, Ang-1, Tie-2, and Endoglin modulated by E2 treatment of MSCs inserted in the Matrigel^®^ plugs. Experiments were performed in triplicate and the results normalized to GAPDH. * *p* < 0.05 versus Control (plugs with MSC only). Values represent the mean ± SEM from three separate experiments conducted in triplicates.

We employed the RT-qPCR technique to confirm the expression of pro-angiogenic genes in response E2. As compared to vehicle treated control plugs (plugs with MSC only), the levels of VEGFR-2, VEGF-A, ANG-1, Tie-2, and Endoglin were up-regulated by 40%, 45%, 95%, 75%, and 30%, respectively, in plugs with E2 treated MSCs. Experiments were performed in triplicate and results normalized to GAPDH (Figure 7).

## 4. Discussion

In the present study, we show that E2 enhances WJ-MSCs induced angiogenesis in vivo. Importantly, using proteomic profiling, we show that the in vivo stimulatory effects of E2 on angiogenesis are accompanied with significant upregulation of pro-tissue regenerative pathways such as mTOR signaling, PDGF receptor signaling network, VEGF and VEGFR signaling network, Glypican 1 network, and Sphingosine 1-phosphate (S1P) pathway, as well as Angiopoietin-1 and Tie-2. These findings, together with our previous observation that E2 increases migration, proliferation, and integration of WJ-MSCs in cardiac slices in an ex vivo setting, suggest that E2 induces pro-tissue regenerative actions and enhances regenerative capability of WJ-MSCs.

Successful regeneration of damaged tissue requires a well-defined cascade of events, including proteolysis of ECM and damaged tissue, generation of growth factors, activation of pro-growth, pro-migration, and chemotactic mechanisms, as well as adhesion molecules [12]. Molecules triggering regenerative actions not only activate local cells, but also attract progenitor or stem cells from within the circulation [12]. E2 significantly modulates many of these mechanisms in WJ-MSCs [15]. In this regard, E2 upregulates: (1) MMPs responsible for proteolytic activity; (2) adhesion molecules (integrins, adherens, and VCAM-1), exosomes, and vesicles which collectively act as paracrine building blocks for tissue repair; (3) growth factors and angiogenesis regulators including VEGF, angiogenin, and NO; (4) chemokines which stimulate cell migration, cell growth, and proangiogenic actions; (5) pathways for growth signaling (MAPK/JAK-STAT/PI3K), cell adhesion, and energy metabolism; (6) transcriptional factors [15]; and (7) circulating progenitor endothelial cells known to induce angiogenesis [25].

Using proteomic profiling, we assessed E2-induced in vivo changes of proteins in MSCs that were associated with E2-induced capillary formation. Proteins play a prominent role in regulating numerous biological and physiological processes in cells, tissues, and organs [13]. Changes in protein expression have specific functions and play crucial roles in regulating tissue/organ growth, development, metabolic regulation, disease progression, and pathophysiology [13]. Moreover, proteins act as signatures and are useful in defining/characterizing the effects of treatments on cells and tissues, including processes such as tissue regeneration. Proteomics deals not only with global proteins, but also their isoforms, thus helping us to understand the different mechanisms employed by cells and organisms [13]. Data obtained from a proteomic analysis can reveal biological and pathophysiological information about drug-cell interactions and effects of treatments. Differentially abundant proteins can serve as therapeutic and diagnostic markers for tissue regeneration. By using advanced proteomic methodologies and sophisticated bioinformatic tools, we can therefore pinpoint alterations in profiles of proteins, their functions, interactions, and structural changes following a pathological stimulus or treatment.

Here, we show that angiogenesis mediated by E2-induced MSCs is accompanied by engagement of signaling pathways including mTOR signaling, the PDGF receptor signaling network, the VEGF-VEGFR signaling network, the Glypican 1 network, and the sphingosine 1-phosphate (S1P) pathway. These findings suggest that these systems are involved in E2-induced tissue regenerative capability. mTOR interacts with other subunits to form two distinct complexes, mTORC1 and mTORC2, which regulate cell survival, growth, migration, and metabolism [26,27]. Importantly, mTORC1 activity is critical for tissue regeneration in multiple organs and contexts; moreover, mTORC1 can induce stemness in mature cells [26,27]. Another network highly regulated by E2-treated MSCs is the PDGFRβ signaling network. Recent studies show that this network regulates cardiomyocyte proliferation and myocardial regeneration [28]. E2 also upregulates the Glypican-1 (a heparan sulfate proteoglycan) and sphingosine 1-phosphate pathways, both of which are mechanosensitive, and protect the heart against pressure overload and mechanical stress and promote intrinsic tissue repair/regeneration [29,30]. Additionally, E2 increases angiogenesis and the proteomic profile for VEGF and VEGFR signaling, suggesting that E2 induces new capillary formation to facilitate tissue regeneration [31]. Indeed, VEGF nanoparticles repair the heart after myocardial infarction [32]. Taken together, the findings of the present study suggest that priming of WJ-MSCs with E2 may promote tissue repair by WJ-MSCs.

We also observe that expressions of angiopoietin-1 (Ang-1) and its receptor Tie-2 are dramatically increased in WJ-MSCs treated with E2. This suggests that the Ang-1/Tie-2 system may critically contribute to the pro-angiogenic and tissue regenerative potential of E2-treated WJ-MSCs. Indeed, it is known that Ang-1 and Tie-2 play a key role in: (1) regulating new vessel formation in the developing heart [33]; (2) promoting cardiac and skeletal survival via integrins [33]; (3) reducing myocardial apoptosis [34]; and (4) promoting progenitor CD133+/c-kit+ cells, which facilitate myocardial healing following infarction [35]. Furthermore, Tie-2 can interact with NOTCH signaling, a signaling network that plays a role in regulating cardiac development and regeneration [36]. Taken together, our observations from proteome profiling and expression studies suggest E2-primed MSCs may facilitate tissue repair via multiple growth regulatory pathways.

E2-induced capillary formation by WJ-MSCs in vivo would facilitate tissue repair following heart damage. In this regard, angiogenesis plays a critical role in restoring perfusion and mitigating hypoxia, processes that promote the repair of infarcted myocardium [37,38]. Enhanced angiogenesis increases cardiomyocyte survival along the endocardium in the ischemic zone and suppresses ventricular remodeling in infarcted hearts [37]. Angiogenesis precedes cardiomyocyte migration and neomyogenesis [38].

Previously we found that treatment with E2, via ERs, increased migration, incorporation, and engraftment of human WJ-MSCs into slices of myocardial tissue, and that this was promoted by mechanisms that repair and remodel tissues [15]. We also observed that E2 treatment of MSCs in vitro augmented the expression of MMP-2, MMP-9, and EMMPRIN, which are proteolytic enzymes that break down/digest extracellular matrix proteins, modulate protein/receptor function, and promote angiogenesis by increasing pro-angiogenic molecules VEGF and angiogenin [15]. The pro-angiogenic and tissue regenerative actions of E2 on WJ-MSCs are also supported by the facts that in vitro E2 upregulates multiple repair mechanisms. These repair mechanisms include not only adhesion molecules (such as integrins, adherens, and VCAM-1) and proteolytic enzymes (such as MMPs), but also exosomes and microvesicles (which promote tissue repair by serving as building blocks). In addition, in vitro E2 increases the expression of regulators of angiogenesis (such as VEGF, angiogenin, and nitric oxide), chemokines (which stimulate cell migration), growth signaling pathways (such as MAPK/JAK-STAT/PI3K) that promote cell adhesion and energy metabolism and transcriptional factors [15]. The present study extends our in vitro observations to the in vivo setting by showing that treatment with E2 in vivo increases: (1) cellular components such as lysosomes (represent 60 hydrolases including cathepsin); (2) exosomes, which are critical for regulated cell repair and dead cell clearance and breakdown for reutilization [39,40]; (3) mTOR signaling; (4) PDGF receptor signaling; (5) glypican 1 network signaling; (6) sphingosine 1-phosphate signaling; and (7) angiopoietin-1/Tie-2 signaling. Importantly, increased capillary formation by WJ-MSCs treated with E2 also strengthens our contention that E2 may enhance MSC-induced tissue repair/regeneration.

Using FunRich, we extracted miRNA-associated data from our proteomic analysis. FunRich is widely used to convert UNIPROT IDs of detected proteins to miRNAs corresponding to each protein/gene [41]. In E2-treated MSCs in vivo, we obtained in silico data consistent with modulation of 321 associated miRNAs that regulate biological processes, molecular functions, and cellular components. These miRNAs are significantly involved in cell communication, signal transduction, cellular metabolism, energy production, and multiple biological processes, including regulation of nucleobase/nucleoside/nucleotide/nucleic acid metabolism. Gene Ontology category analysis revealed a prominent effect on transcription factor activity. The cellular component miRNAs are largely involved in regulating events in the nucleus, cytoplasm, Golgi apparatus, lysosomes, cytosol, endoplasmic reticulum, plasma membrane, exosomes, and mitochondria. The consistency in miRNA and protein-based targets in MSCs treated with E2 reaffirms the importance of pathways influenced by E2 with regard to E2’s angiogenic and tissue repair actions.

Heart disease is the leading cause of mortality in women. A permanent cure for damaged hearts remains elusive due to the scarcity of donor hearts and transplant failures due to mismatch. This highlights the need for alternative therapeutic strategies [1,14]. The use of adult MSCs exhibiting pluripotent characteristics is of immense therapeutic interest for treating heart damage following myocardial infarction, coronary artery disease, and reperfusion injury following ischemia. Importantly, MSCs or MSCs-derived molecules may provide the only viable alternative to heart transplantation as a permanent cure for heart damage [2,9,42,43,44]. Findings from animal and human studies suggest that MSCs are effective in repairing heart damage and improving function. A major advantage of MSCs is that MSCs can be isolated from multiple tissues in adults and are relatively easy to culture into desired cell types. Another advantage is that there are fewer ethical concerns with regard to using MSCs versus embryonic cells. Consequently, MSCs have become the cell of choice for therapeutic use in tissue repair [5]. However, a review of studies conducted in the last two decades suggests that a limiting factor for MSC-based therapy is the engraftment of low number of MSCs (0.44–1%) [3,10] in damaged sites and lack of understanding of the mechanisms involved in MSCs engraftment [11]. There is a consensus that strategies that would improve MSCs homing and engraftment are required to optimize their therapeutic use. Our finding that treatment of MSCs with E2 significantly induces capillary formation in vivo, together with our previous observation that E2 improves MSC engraftment in cardiac slices and stimulates their tissue directed migration [15], suggests that priming of MSCs prior to delivery would increase their therapeutic capability to repair damaged tissue.

E2 facilitates the release of progenitor ECs (PECs) and promotes cardiovascular repair by enhancing their engraftment, angiogenesis, and function [25,45]. However, compared to PECs, the pleotropic nature of MSCs renders MSCs a more attractive and viable option for tissue repair. The advantages of Wharton’s jelly derived MSCs are numerous and include: (1) induction of scar-less wound healing; (2) superior plasticity properties; (3) greater expansion capability; (4) unique immunologic characteristics; (5) low risk of rejection upon allogeneic transplantation; (6) low risk, compared to embryonic stem cells, of teratoma formation [7,46,47]; (7) multipotent properties between embryonic and adult stem cells [6,7]; (8) unique developmental and operational characteristics; (9) ease of isolation; and (10) obtainable from a tissue usually considered residual and often discarded. Although WJ-MSCs are clearly viable for tissue repair [6,7,8,48], MSCs from other sources (bone marrow, umbilical cord, adipose tissue, and embryonic cells) also possess repair capability and may be useful for tissue repair.

There are sex dimorphisms in the paracrine functions of MSCs. For example, infusion of female, compared with male, MSCs induces a better post-ischemic myocardial functional recovery [19]. Likewise, female, compared to male, bone marrow derived MSCs secrete less pro-inflammatory cytokines and more VEGF [19,20]. As MSCs produce E2 [47], synthesis of E2 at the site of injury may play a role in regulating paracrine factors that initiate tissue repair by surrounding cells including ECs/PECs. Our findings suggest that E2-primed MSCs express enhanced heart repairing capability; however, we have also observed a similar improvement in the migration and integration of MSCs in cardiac slices in response to androgens [48]. As sex plays an important role in the pathophysiology of cardiovascular disease and heart transplantation, priming of MSCs with respective sex steroids may provide a simple therapeutic approach to enhance repair of heart damage.

Biochemical interactions between multiple cell types mediate both tissue repair and remodeling [5,12]. Often, tissue repair and remodeling begin with proteolysis of extracellular matrix (ECM). Subsequently, adhesion molecules allow for recruitment and engraftment of pluripotent MSCs. Release of growth and pro-angiogenesis factors then promote angiogenesis and proliferation of both recruited and existing cells [5]. MMPs are a group of enzymes that degrade the ECM and basement membranes, processes that produce and activate latent receptors and growth factors. This orchestrates the initiation of multiple cellular events, such as cell–cell adhesions and cell migration, invasion, proliferation, and apoptosis, resulting in tissue remodeling [49,50,51,52]. MMP substrates include angiogenesis factors, growth factors, cell adhesion molecules, receptors, cytokines, as well as ECM and basement membrane molecules [51,52]. Resident cells of tissues do not synthesize MMPs unless MMPs are required for remodeling [53]. For example, MMP-2 and MMP-9 levels are spatiotemporal modulated after a myocardial infarction or end stage heart failure, and play important roles resulting in tissue remodeling [54]. Although MMP-2 and MMP-9 possess gelatinase activity and display considerable overlap in substrates, selective differences in their modes of action exist. For example, compared to MMP-2, MMP-9 is incapable of direct proteolysis of collagen-I [51]. MMPs are regulated by paracrine factors generated following injury [50,51,55,56].

Our proteomic screening data provides evidence that in WJ-MSCs E2 upregulates lysosomes, which are acidic organelles central for cellular degradation, repair of the plasma membrane, exocytosis, cholesterol homoeostasis, and regulated cell death. Among lysosomal hydrolases, cathepsins are most abundant, and play an important role in heart pathophysiology as well as cardiac repair and remodeling post-infarction [39,40]. These findings are consistent with our previous observations in which in vitro treatment of WJ-MSCs with E2 induced biological processes essential for removing scar tissue, promoting stem cell attachment, and engraftment and encouraging tissue repair. These biological processes involve not only proteolysis and apoptosis, but also cell adhesion and production of anchoring junctions and mRNA synthesis.

Data from the present study provide strong evidence that in WJ-MSCs in vivo E2 induces exosome and/or extracellular vesicles. Exosomes and extracellular vesicles (EVs) play a major role in tissue repair by functioning as carriers, to both local and remote cellular destinations, and provide cargo such as proteins; small, long, and non-coding RNAs; and lipids. E2 induces exosome formation [15], and MSC-derived exosomes induce cardiac repair and reduce scar tissue [47]. Taken together, our proteomics data support our overall findings that pretreatment of MSCs with E2, in vivo, may facilitate pro-repair mechanisms to enhance MSC engraftment.

The fact that E2 significantly induced EV proteins in MSCs suggests that EVs may play a role in MSC-induced angiogenesis in the presence of E2. EVs are membranous structures of different sizes with biological activity [57]. They encompass exosomes (40–140 nm), microvesicles (50–1000 nm), and apoptotic bodies (1–5 µm) [57]. The cargo within EVs varies and is tissue, cell, and culture condition dependent. EVs can mediate site-specific actions, including tissue repair, by regulating apoptosis, cell proliferation, angiogenesis, and inflammation [57]. Recent studies provide evidence that MSC-derived EVs are effective in inducing beneficial effects in inflammation and tissue repair [57]. Studies show that MSC derived EVs protect against myocardial-ischemia reperfusion injury [58] with a potency equivalent to MSCs and MSC-conditioned medium [57,58]. MSC-derived EVs protect against myocardial injury by suppressing cell apoptosis and scar formation, and by improving endothelial cell health in the microvasculature [59] via pro-survival pathways [59]. Interestingly, the human WJ-MSCs secretome induces angiogenesis, and has a more complete angiogenic network with higher concentrations of angiogenesis-related proteins compared to human bone marrow MSCs [60]. MSC-derived EVs contain and carry growth and angiogenic factors (e.g., VEGF, PDGF, Ang1, Endoglin, HGF, and SDF-1) which likely contribute to tissue repair and angiogenic actions [57]. E2 induces EV formation in breast cancer cells [61], and E2 primed MSCs are more protective against myocardial recovery after ischemia [19]. These findings, together with our observation that exosome-associated proteins are significantly increased in E2 plus MSC implants, suggest that increased angiogenesis is mediated, in part, via E2-stimulated release of EVs from MSCs. However, this possibility needs to be confirmed. If true, E2 may serve as a stimulator of proangiogenic EV regulator in MSCs.

Apoptotic cells generate EVs with tissue repairing capability [62,63]. Here, we observed significant upregulation of both the intrinsic and extrinsic apoptotic signaling pathways. It is feasible that E2 may facilitate phagocytotic activity to clear dead cells and facilitate formation of apoptotic EVs. Interestingly, in heart injury, transplanted apoptotic EVs derived from MSCs regulate autophagy in cardiac endothelial cells and improve cardiac functional recovery in a myocardial infarction model [62,63]. Taken together, the above findings strongly suggest that MSC-derived EVs may contribute to the increased angiogenesis in plugs with MSCs plus E2. This possibility needs to further investigated. If EVs are indeed as potent as MSCs, injecting them for therapeutic heart repair would be more practical and with little or no deleterious effects.

We identified several limitations to this study. Although our in vivo findings provide evidence that E2 promotes capillary formation by WJ-MSCs, proof of its effectiveness and role in tissue (heart) repair and regeneration need further validation. Whether E2 induces beneficial or deleterious actions on the cardiovascular system is still unclear. MSCs can evolve into many different cell types, including bone cells; thus, MSCs could induce calcification within blood vessels. Additionally, E2-treated MSCs may, via capillary formation, render coronary plaques unstable and thus susceptible to rupture. Upregulation of MMP-2, MMP-9, and EMMPRIN, which are lysosomal components, is associated with deleterious effects following cardiac reperfusion injury, whereas their inhibition is protective.

The rationale for using 100 nmol/L of E2 was based on the fact that E2 levels are high during reproductive tissue remodeling during ovulation and pregnancy. The follicular fluid levels in oocytes during ovulation can range from 100 to > 1000 nmol/L. As our goal was to assess whether priming of MSCs with E2 induces pro-repair molecules/pathways, we elected to use 100 nmol/L for our experiments. Hence, the concentrations in the present study represent levels reflected during tissue remodeling, and not circulating E2 levels (in the range of 1 nmol/L). Our contention is supported by findings from Erwin and coworkers, who demonstrated that MSCs treated with supra-physiologic E2 concentrations improved myocardial recovery after ischemia [19].

The clinical potential of our findings is evident in this pilot study. In this context, E2 may be of therapeutic relevance in enhancing WJ-MSC-induced capillary formation to promote cardiac repair. The present findings, along with our previous observations that E2-induced WJ-MSCs integrate in cardiac slices, that E2 activates ECs and progenitor cells, and that MSCs can transform into cardiomyocytes, suggest that E2 priming of WJ-MSCs may amplify the chances of their success to repair a damaged heart. However, additional in vivo studies are necessary to confirm this possibility.

Here, we provide the first evidence that in vivo E2 enhances WJ-MSC-induced angiogenesis. Proteomic analysis demonstrates that the stimulatory effects of E2 are associated with over-representation of proteins related to cellular components, biological processes, biological pathways, and molecular pathways. Importantly, in MSCs E2 induces upregulation of several pro-tissue regenerative signaling pathways, including mTOR signaling, PDGF receptor signaling, VEGF and VEGFR signaling, Glypican 1 signaling, and sphingosine 1-phosphate signaling, as well as Angiopoietin-1 and Tie-2 signaling. These findings strongly suggest that E2 induces MSCs pro-tissue regenerative actions. This is further supported by the fact that E2 enhances MSC-induced angiogenesis. Although we did not measure damaged tissue repair, we have previously shown that, in an ex vivo setting, E2 increases the integration of MSCs into cardiac slices. Proteomic screening of MSCs provides evidence for E2-mediated increases in key molecules, cellular structures, and tissue remodeling processes including hydrolases, exosomes/vesicles, apoptosis, proteolysis, angiogenesis, cell adhesion, integrins, migration, and growth. Taken together, our findings suggest that priming of MSCs with E2 may functionally increase their capability to repair and regenerate cardiac tissue. As E2 is known to increase proteolytic enzymes (MMPs) as well as proangiogenic/tissue regenerative molecules, we speculate that E2 may sequentially promote clearing of dead/scar tissue and promote growth/angiogenesis in a paracrine fashion. If true, priming of MSCs with sex steroids would provide a simple and effective way to enhance MSC-mediated heart repair in a gender specific manner. Additionally, priming of MSCs with E2 may be a novel approach to improve outcomes in women undergoing heart transplantation.

## Figures and Tables

**Table 1:** Top ten downregulated proteins by Estradiol in MSCs and extracted using Proteome Discoverer Software.

Uniprot Code	Protein Description	Ratio (Estradiol Treatment/Control)	Standard Deviation	*p*-Value
Q2KJY2	Kinesin-like protein KIF26B	0.072322	0.025708	1.16 × 10^−7^
P35555	Fibrillin-1	0.076573	0.018242	9.90 × 10^−20^
P02462	Collagen alpha-1(IV) chain	0.10606	0.039357	1.36 × 10^−9^
P01023	alpha-2-macroglobulin	0.16071	0.065218	8.08 × 10^−10^
P27797	Calreticulin	0.16071	0.065218	8.08 × 10^−10^
P68133	Actin, alpha skeletal muscle	0.20209	0.11097	2.75 × 10^−5^
Q92600	CCR4-NOT transcription complex subunit 9	0.23116	0.11491	1.93 × 10^−5^
Q13867	bleomycin hydrolase	0.44919	0.1537	1.30 × 10^−8^
O43676	NADH dehydrogenase (ubiquinone) 1 beta subcomplex subunit 3	0.46305	0.18816	1.09 × 10^−6^
P60903	Protein S100-A10	0.52898	0.1872	2.25 × 10^−6^

**Table 2:** Top ten upregulated proteins by Estradiol in MSCs and extracted using Proteome Discoverer Software.

Uniprot Code	Protein Description	Ratio (Estradiol Treatment/Control)	Standard Deviation	*p*-Value
Q15149	plectin	1.5706	0.79404	0.00057
P08133	annexin A6	1.6495	0.70015	0.00075
P38646	Stress-70 protein, mitochondrial	2.3358	0.81957	3.49 × 10^−12^
P46926	glucosaminx10-6-phosphate isomerase 1	2.8277	1.1221	6.49 × 10^−7^
O43497	Voltagx10-dependent T-type calcium channel subunit alpha-1G	3.1222	0.95104	1.76 × 10^−7^
Q9ULV0	unconventional myosin-Vb	3.1632	1.334	1.91 × 10^−6^
Q9NZM1	Myoferlin	3.5759	1.8188	7.38 × 10^−7^
O95571	Persulfide dioxygenase ETHE1, mitochondrial	4.6351	1.8729	6.26 × 10^−7^
Q9NP66	High mobility group protein 20A	5.6817	2.4269	3.18 × 10^−7^
Q14865	AT-rich interactive domain-containing protein 5B	8.6372	3.5807	4.41 × 10^−7^
P05997	Collagen alpha-2(V) chain	8.6372	3.5807	4.41 × 10^−7^
O75794	Cell division cycle protein 123 homolog	9.5802	5.5167	2.49 × 10^−7^
Q86VB7	Scavenger receptor cysteinx10-rich type 1 protein M130	13.854	5.8186	1.36 × 10^−9^

## Data Availability

The proteomic data is deposited at the ProteomeXchange consortium via PRIDE partner repository with dataset identifier PXD026380 and 10.6019/PXD026380. For all other data, contact the authors.

## Supplementary Materials

The following are available online at https://www.mdpi.com/article/10.3390/cells10092181/s1, Figure S1: MSCs express estrogen receptors and estradiol modulates hormone associated cellular mechanisms, Figure S2: Map of detected Uniprot ID’s statistically analyzed by Reactome software showing significant up-regulation of proteins in specific cellular homeostasis by estradiol in MSCs, Figure S3: FunRich analysis of genes and their site of expression, Table S1: Information on Major Resources Used, Table S2: Primers used for qRT-PCR assay, Table S3: Funrich Analysis of Over-represented Proteins of the Cellular Component in MSCs following estradiol treatment, Table S4: Funrich Analysis of Over-represented Proteins of the Molecular Function in MSCs following estradiol treatment, Table S5: Funrich Analysis of Over-represented Proteins of the Biological Process in MSCs following estradiol treatment.
