# Supplementary material for: Proteomic Analysis of Estrogen-Mediated Enhancement of Mesenchymal Stem Cell-Induced Angiogenesis In Vivo
Source: Cells. 2021 Aug 24;10(9):2181. doi: 10.3390/cells10092181 (PMC8468955; doi:10.3390/cells10092181)
Supplement: Supplementary file 1 [file cells-10-02181-s001.zip › cells-1301180-SI.pdf]

Supplementary materials

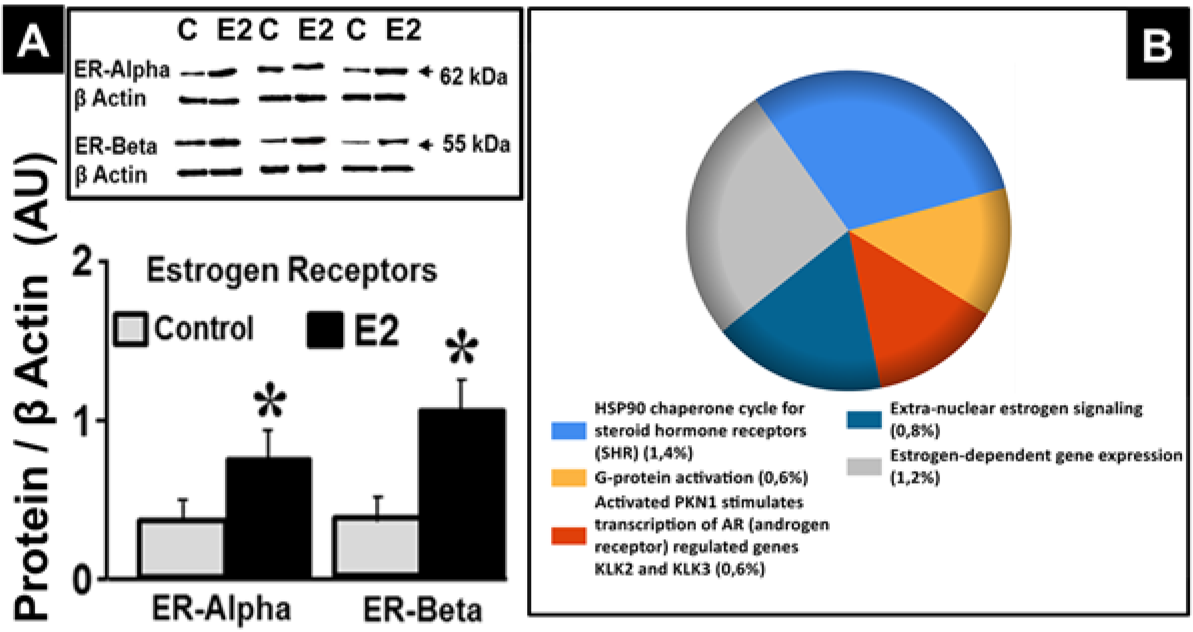

Figure S1 MSCs express estrogen receptors and estradiol modulates hormone associated cellular mechanisms. (**A**) Western Blots depicting the presence of estrogen receptors (ER) alpha and ER Beta in WJ-MSCs in presence and absence of E2 and as previously described by us (Mihai et al., J Mol Cell Cardiol 2019, 133, 115-124, doi: 10.1016/j.yjmcc.2019.06.007). (**B**) Depicts different percentages (%) of hormone associated cellular mechanisms in E2 treated MSCs. FunRich (Uniprot database) analysis of the proteins differentially expressed by E2 stimulation showed a significant over-representation (False discovery rate corrected p-value < 0.05) of proteins participating in different cellular components and identified by Reactome pathways to reveal the different percentages (%) of hormone associated cellular mechanisms in E2 treated MSCs.

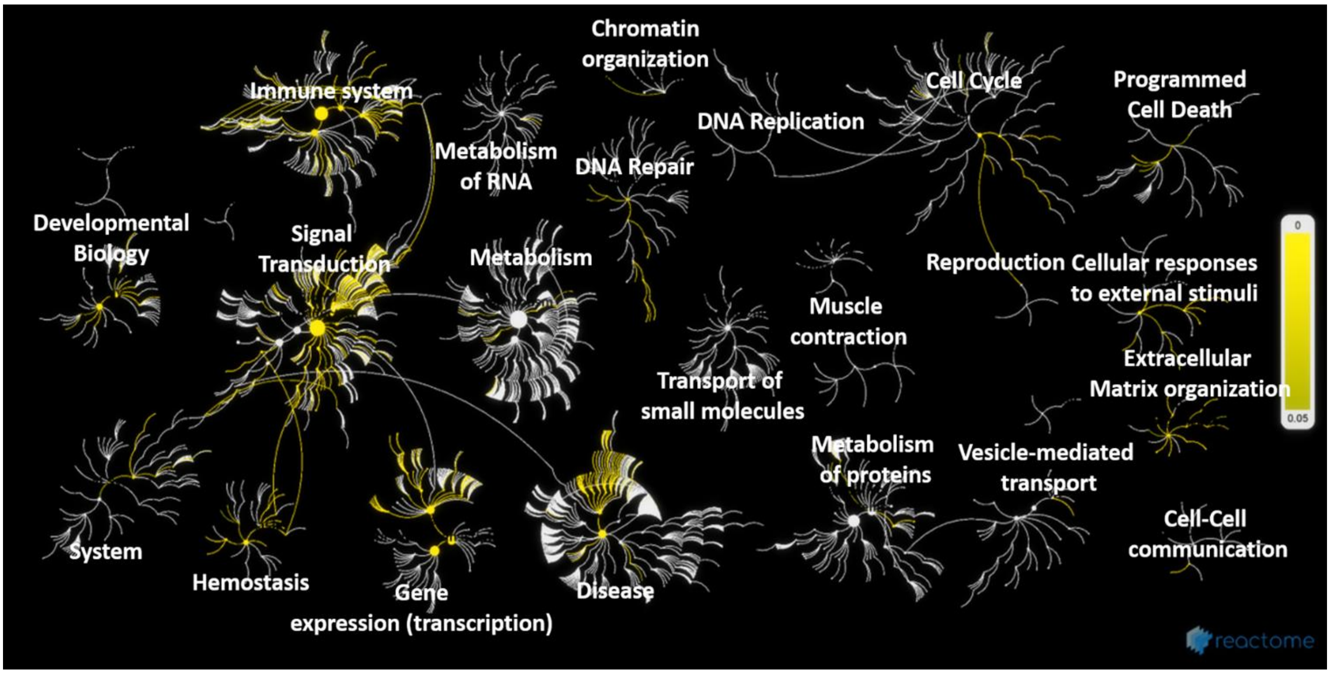

Figure S2 Map of detected Uniprot ID's statistically analyzed by Reactome software using p-value < 0.05 and showing up-regulation of proteins in specific cellular homeostasis.

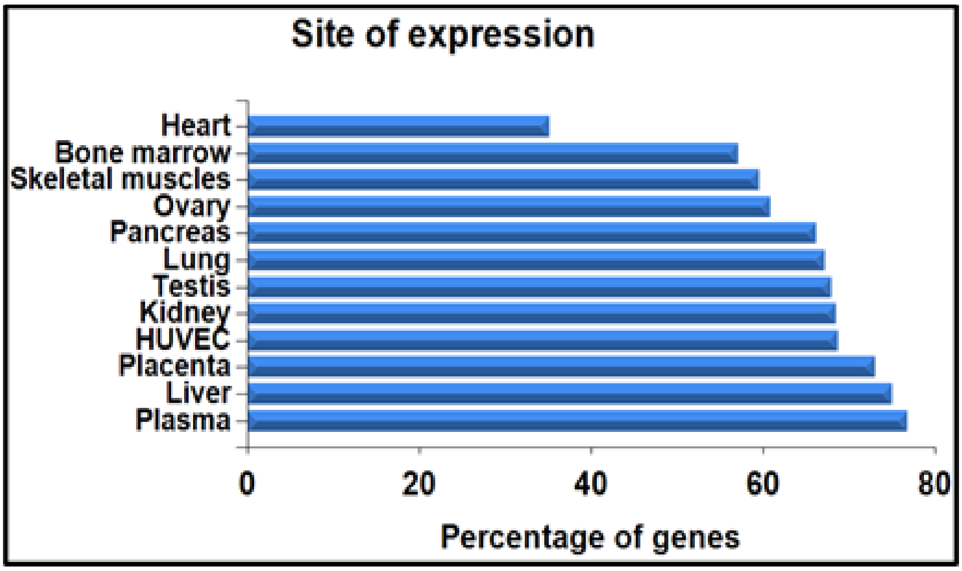

Figure S3 *FunRich analysis of genes and their site of expression.* Tissue location of enriched expression for targets determined using FunRich v.3.0, which infers regional and cell type enrichment of target mRNAs for a given list of RNAs.

Supplementary Table-1: Information on Major Resources Used

Animals used in *in vivo* studies

Species	Vendor or Source	Background Strain
Mus musculus	The Jackson Laboratory	C57Bl/6

Animal breeding

	Species	Vendor or Source	Background Strain	Other Information	Species	Vendor or Source	Background Strain
**Parent - Male**	Mus musculus	The Jackson Laboratory	C57Bl/6	Bred and maintained in SPF conditions	Mus musculus	The Jackson Laboratory	C57Bl/6
**Parent - Female**	Mus musculus	The Jackson Laboratory	C57Bl/6	Bred and maintained in SPF conditions			

Human Cultured Cells – MSCs isolated and cultured from Wharton's Jelly

Proteomic Analysis Reagents

Name	Vendor or Source
Ethylenediaminetetraacetic acid (EDTA), acetone, formic acid, acetonitrile, sodium deoxycholate (DOC), ammonium bicarbonate, DL-dithiothreitol (DTT),	Sigma-Aldrich, Missouri, USA

iodoacetamide (IAA), trizma hydrochloride (Tris-HCl), N-acetyl-L-cysteine (NAC), urea and water	Promega (Wisconsin, USA)
Trypsin (Gold grade)	Roche (Indiana, USA)
Protease Inhibitor Complete Cocktail	Waters (Massachusetts, USA).
C18 columns for solid phase extraction	Tebu-Bio, Cytoskeleton, Colorado, USA
Advanced Protein Assay ADV-01A	

Supplementary Table-2: Primers used for qRT-PCR assay

Primer (human)	Forward primer (5′–3′)	Reverse primer (5′–3′)
Ang-1	CAGGAGGATGGTGGTTTGATG	TGGTTTTGTCCCGCAGTATAGAA
Tie-2	GATTTTGGATTGTCCCGAGGTCAAG	CACCAATATCTGGGCAAATGATGG
Endoglin	CAATGCCAGCATTGTCACCTCC	AGAGGCTGTCCATGTCGATGCA
VEGF	GCTACTGCCATCCAATCGAG	TCTTTCTTTGGTCTGCATTCAC
VEGFR-2	AAGTAATCCCAGATGACAACCA	CCTTCAGATGCCACAGACTC
GAPDH	GTTTCTATAAATTGAGCCCGCAG	CGACCAAATCCGTTGACTCC

Supplementary Table-3: FunRich Analysis of Over-represented Proteins of the Cellular Component in MSCs following estradiol treatment

CELLULAR COMPONENT	NO. OF PROTEINS IN THE DATASET	PERCENTAGE OF PROTEINS	FOLD ENRICHMENT	P-VALUE

PLASMA MEMBRANE	359	41,07551487	1,631479137	3,69927E-26
CYTOSOL	316	36,15560641	1,275540127	1,72999E-07
CYTOPLASM	262	29,9771167	1,185615856	0,00073998
NUCLEUS	242	27,68878719	0,966772797	0,749718916
EXTRACELLULAR EXOSOME	231	26,43020595	2,342427559	2,4204E-37
NUCLEOPLASM	178	20,36613272	1,004858387	0,484778077
EXTRACELLULAR REGION	171	19,56521739	1,876003107	1,22736E-16
MEMBRANE	164	18,76430206	1,667814466	1,53755E-11
EXTRACELLULAR SPACE	164	18,76430206	2,131828351	2,25795E-21
INTEGRAL COMPONENT OF PLASMA MEMBRANE	159	18,19221968	2,399110068	4,49029E-26
INTEGRAL COMPONENT OF MEMBRANE	127	14,53089245	0,729924039	0,999990136
MITOCHONDRION	81	9,267734554	1,342016312	0,004024259
INTRACELLULAR MEMBRANE-BOUNDED ORGANELLE	73	8,352402746	1,787811029	1,00002E-06
CELL SURFACE	73	8,352402746	2,681701132	1,17538E-14
GOLGI APPARATUS	70	8,009153318	1,509601246	0,000373306
PERINUCLEAR REGION OF CYTOPLASM	61	6,979405034	1,859435612	2,29834E-06
ENDOPLASMIC RETICULUM	57	6,52173913	1,172331952	0,118479301
PROTEIN-CONTAINING COMPLEX	55	6,292906178	1,842643031	9,46416E-06
COLLAGEN-CONTAINING EXTRACELLULAR MATRIX	54	6,178489703	3,335078052	5,18435E-15
ENDOPLASMIC RETICULUM MEMBRANE	50	5,720823799	1,099492076	0,261299725
CHROMATIN	49	5,606407323	1,086476417	0,292414282
EXTERNAL SIDE OF PLASMA MEMBRANE	47	5,377574371	2,743778171	3,3605E-10
SYNAPSE	46	5,263157895	2,462758109	1,65524E-08
FOCAL ADHESION	44	5,034324943	2,29796849	2,51025E-07
APICAL PLASMA MEMBRANE	40	4,576659039	2,456348986	1,5329E-07
NEURON PROJECTION	40	4,576659039	2,680348611	1,37634E-08
ENDOPLASMIC RETICULUM LUMEN	40	4,576659039	2,813034757	3,42368E-09
BASOLATERAL PLASMA MEMBRANE	39	4,462242563	3,901570566	2,12748E-13
DENDRITE	38	4,347826087	1,994456862	4,28543E-05
NEURONAL CELL BODY	38	4,347826087	2,453772403	3,21393E-07

MEMBRANE RAFT	36	4,118993135	3,335338462	1,92058E-10
MITOCHONDRIAL MATRIX	36	4,118993135	2,034856369	4,46488E-05
CYTOSKELETON	33	3,775743707	1,670385153	0,002858631
GOLGI MEMBRANE	33	3,775743707	1,204172622	0,15578046
BLOOD MICROPARTI-CLE	32	3,661327231	4,802089554	8,20547E-14
NUCLEOLUS	32	3,661327231	0,818651322	0,901571894
CYTOPLASMIC VESI-CLE	31	3,546910755	2,334336674	1,07545E-05
LYSOSOMAL MEM-BRANE	30	3,432494279	1,99786415	0,000258353
GLUTAMATERGIC SYNAPSE	28	3,203661327	1,969335255	0,00051457
ENDOSOME	26	2,974828375	1,94421264	0,000968804
AXON	25	2,860411899	1,764232737	0,004421972
LYSOSOME	25	2,860411899	1,988044957	0,000875804
MITOCHONDRIAL IN-NER MEMBRANE	24	2,745995423	1,275558748	0,133361557
NUCLEAR MEMBRANE	23	2,631578947	2,09481517	0,000681261
MITOCHONDRIAL OUTER MEMBRANE	22	2,517162471	2,664073532	2,72443E-05
SECRETORY GRANULE LUMEN	22	2,517162471	4,077067928	1,62045E-08
ACTIN CYTOSKELE-TON	22	2,517162471	2,021049017	0,001408814
POSTSYNAPTIC MEM-BRANE	21	2,402745995	3,266910933	1,64352E-06
CELL CORTEX	21	2,402745995	3,174239181	2,64308E-06
NUCLEAR BODY	20	2,288329519	1,340509265	0,113443136
CELL-CELL JUNCTION	20	2,288329519	2,492809493	0,000157469
RECEPTOR COMPLEX	20	2,288329519	2,039593088	0,002040256
CENTROSOME	19	2,173913043	0,803538096	0,866465355
POSTSYNAPTIC DEN-SITY	19	2,173913043	1,701572354	0,017110058
CLATHRIN-COATED ENDOCYTIC VESICLE MEMBRANE	19	2,173913043	5,78476269	3,11215E-10
EXTRACELLULAR MA-TRIX	19	2,173913043	1,646238917	0,023272424
ADHERENS JUNCTION	18	2,059496568	2,383001804	0,000570237
CAVEOLA	18	2,059496568	5,480461654	2,37491E-09
FICOLIN-1-RICH GRANULE LUMEN	18	2,059496568	3,094001455	1,93054E-05
ENDOSOME MEM-BRANE	18	2,059496568	1,675416446	0,022885963
VESICLE	17	1,945080092	2,337804044	0,000995228
LATERAL PLASMA MEMBRANE	17	1,945080092	5,939731272	1,7095E-09

ANCHORED COMPONENT OF MEMBRANE	17	1,945080092	3,587595336	4,35362E-06
DENDRITIC SPINE	17	1,945080092	2,399728527	0,000740813
Z DISC	16	1,830663616	2,818600854	0,000170623
NUCLEAR SPECK	16	1,830663616	0,833913326	0,806986749
MELANOSOME	15	1,71624714	3,165773427	7,16038E-05
CELL JUNCTION	15	1,71624714	1,614942548	0,046636077
SECRETORY GRANULE MEMBRANE	15	1,71624714	3,26267497	5,01168E-05
SARCOLEMMA	15	1,71624714	3,365695967	3,45094E-05
MICROVILLUS	15	1,71624714	4,844338341	2,98659E-07
LAMELLIPODIUM	14	1,601830664	1,667340698	0,042760649
POSTSYNAPSE	14	1,601830664	3,279536956	8,36431E-05
EARLY ENDOSOME	14	1,601830664	1,139157507	0,347820517
NUCLEAR ENVELOPE	14	1,601830664	1,676707254	0,041109444
SPECIFIC GRANULE MEMBRANE	14	1,601830664	3,279536956	8,36431E-05
MITOCHONDRIAL MEMBRANE	13	1,487414188	2,368742354	0,003349575
ENDOCYTIC VESICLE MEMBRANE	13	1,487414188	4,016324342	1,65191E-05
CELL PROJECTION	13	1,487414188	2,199559899	0,006295037
PRESYNAPSE	12	1,372997712	2,368877209	0,004725399
TRANS-GOLGI NETWORK MEMBRANE	12	1,372997712	2,584207932	0,002295766
PLATELET ALPHA GRANULE LUMEN	12	1,372997712	3,818272307	5,8629E-05
TRANSCRIPTION REGULATOR COMPLEX	12	1,372997712	1,325643372	0,197098051
T-TUBULE	12	1,372997712	6,090512433	3,18585E-07
EARLY ENDOSOME MEMBRANE	12	1,372997712	1,541247077	0,091400199
LYSOSOMAL LUMEN	12	1,372997712	2,721651179	0,001467589
CATENIN COMPLEX	12	1,372997712	8,525905609	4,19757E-09
EXTRACELLULAR VESICLE	12	1,372997712	4,651198461	7,19838E-06
PEROXISOME	11	1,258581236	2,212605526	0,010985727
TERTIARY GRANULE MEMBRANE	11	1,258581236	3,212687465	0,000566622
CYCLIN-DEPENDENT PROTEIN KINASE HOLOENZYME COMPLEX	11	1,258581236	7,816005058	5,50228E-08
BASAL PLASMA MEMBRANE	10	1,14416476	4,26423108	9,15579E-05
APICAL PART OF CELL	10	1,14416476	3,331576258	0,000746245
RECYCLING ENDOSOME	10	1,14416476	1,692359307	0,072590643
GROWTH CONE	10	1,14416476	1,57954371	0,103101942

BASEMENT MEMBRANE	10	1,14416476	2,538438237	0,005873203
MIDBODY	10	1,14416476	1,300251182	0,242177495
VOLTAGE-GATED CALCIUM CHANNEL COMPLEX	10	1,14416476	7,895379352	2,00706E-07
BICELLULAR TIGHT JUNCTION	9	1,029748284	1,654599438	0,095263175
CYTOPLASMIC VESICLE MEMBRANE	9	1,029748284	1,351665944	0,223752527
SPECIFIC GRANULE LUMEN	9	1,029748284	3,095469775	0,002301617
TERTIARY GRANULE LUMEN	9	1,029748284	3,489367038	0,000966057
HIGH-DENSITY LIPOPROTEIN PARTICLE	9	1,029748284	7,994588954	7,33781E-07
SYNAPTIC VESICLE	9	1,029748284	1,573232364	0,119995005
TERMINAL BOUTON	9	1,029748284	3,916549292	0,000401302
LATE ENDOSOME	9	1,029748284	1,380836492	0,20635112
RUFFLE MEMBRANE	9	1,029748284	2,086187162	0,028616054
ACETYLCHOLINE-GATED CHANNEL COMPLEX	9	1,029748284	11,98938668	9,0232E-09
EXTRINSIC COMPONENT OF CYTOPLASMIC SIDE OF PLASMA MEMBRANE	9	1,029748284	2,864499042	0,003940688
TRANS-GOLGI NETWORK	9	1,029748284	1,09679493	0,438147336
FICOLIN-1-RICH GRANULE MEMBRANE	9	1,029748284	3,146206864	0,002051179
CHROMOSOME	9	1,029748284	0,954928017	0,605359635
SODIUM:POTASSIUM-EXCHANGING ATPASE COMPLEX	9	1,029748284	15,9825213	2,05803E-10

Supplementary Table-4: FunRich Analysis of Over-represented Proteins of the Molecular Function in MSCs following estradiol treatment

---

MOLECULAR FUNCTION	NO. OF PROTEINS IN THE DATASET	PERCENTAGE OF PROTEINS	FOLD ENRICHMENT	P-VALUE
IDENTICAL PROTEIN BINDING	157	18,47058824	1,836811434	1,29144E-14
ATP BINDING	124	14,58823529	1,566971806	2,19995E-07
METAL ION BINDING	95	11,17647059	0,757664215	0,99924305
PROTEIN HOMODIMERIZATION ACTIVITY	72	8,470588235	1,965224811	2,62774E-08
ZINC ION BINDING	63	7,411764706	1,406204203	0,003787567
CALCIUM ION BINDING	61	7,176470588	1,585959553	0,000234627
PROTEIN KINASE BINDING	57	6,705882353	2,291768997	3,81048E-09
RNA BINDING	49	5,764705882	0,660538368	0,999638849
SIGNALING RECEPTOR BINDING	45	5,294117647	2,314187884	1,29467E-07
DNA-BINDING TRANSCRIPTION FACTOR ACTIVITY, RNA POLYMERASE II-SPECIFIC	44	5,176470588	0,651144603	0,999539221
DNA BINDING	42	4,941176471	0,871697031	0,846574645
RNA POLYMERASE II CIS-REGULATORY REGION SEQUENCE-SPECIFIC DNA BINDING	42	4,941176471	0,663201291	0,998991018
PROTEIN HETERODIMERIZATION ACTIVITY	40	4,705882353	2,394442256	2,63335E-07
PROTEIN-CONTAINING COMPLEX BINDING	38	4,470588235	2,044651941	2,28124E-05
ENZYME BINDING	36	4,235294118	1,851453137	0,000277922
SEQUENCE-SPECIFIC DOUBLE-STRANDED DNA BINDING	36	4,235294118	1,214189145	0,131887415
DNA-BINDING TRANSCRIPTION FACTOR ACTIVITY	32	3,764705882	1,347918858	0,053219234
TRANSCRIPTION FACTOR BINDING	31	3,647058824	1,9631335	0,00026308
CHROMATIN BINDING	31	3,647058824	1,36446155	0,049434855
PROTEIN SERINE KINASE ACTIVITY	29	3,411764706	1,495679932	0,020143717
PROTEIN THREONINE KINASE ACTIVITY	29	3,411764706	1,495679932	0,020143717
CADHERIN BINDING	28	3,294117647	1,703678567	0,004233932
DNA-BINDING TRANSCRIPTION ACTIVATOR ACTIVITY, RNA POLYMERASE II-SPECIFIC	27	3,176470588	1,163699786	0,233584181
PROTEIN SERINE/THREONINE KINASE ACTIVITY	26	3,058823529	1,629969397	0,009939635
ACTIN FILAMENT BINDING	26	3,058823529	2,408423514	2,90682E-05
G PROTEIN-COUPLED RECEPTOR ACTIVITY	25	2,941176471	0,651957923	0,993456604
GTP BINDING	25	2,941176471	1,228211595	0,168942863
GROWTH FACTOR ACTIVITY	25	2,941176471	2,873307058	1,7735E-06
PROTEIN KINASE ACTIVITY	23	2,705882353	2,109644887	0,000585252

CALMODULIN BINDING	23	2,705882353	2,162916058	0,000410759
ACTIN BINDING	21	2,470588235	1,41169467	0,070730494
ATPASE ACTIVITY	21	2,470588235	1,515652652	0,038310574
GTPASE ACTIVITY	20	2,352941176	1,237303766	0,192216393
UBIQUITIN PROTEIN LIGASE BINDING	19	2,235294118	1,20756313	0,229734156
PROTEIN C-TERMINUS BINDING	18	2,117647059	1,718964867	0,017783006
TRANSMEMBRANE SIGNALING RECEPTOR ACTIVITY	18	2,117647059	2,019247869	0,003576231
SIGNALING RECEPTOR ACTIVITY	18	2,117647059	1,581129847	0,037022138
ION CHANNEL BINDING	17	2	2,416629302	0,000647186
PROTEASE BINDING	17	2	3,165709477	2,18347E-05
CHAPERONE BINDING	17	2	3,103642827	2,8482E-05
RNA POLYMERASE II TRANSCRIPTION REGULATORY REGION SEQUENCE-SPECIFIC DNA BINDING	17	2	0,959372761	0,606720621
CYTOKINE ACTIVITY	17	2	1,702073033	0,022719701
TRANSCRIPTION REGULATORY REGION SEQUENCE-SPECIFIC DNA BINDING	17	2	1,522054732	0,056158194
HEPARIN BINDING	16	1,882352941	1,805889769	0,016132492
INTEGRIN BINDING	16	1,882352941	2,083699537	0,004294788
TRANSCRIPTION COACTIVATOR ACTIVITY	16	1,882352941	1,267988047	0,197233254
LIPID BINDING	16	1,882352941	2,174949791	0,002799927
PROTEIN DOMAIN SPECIFIC BINDING	15	1,764705882	1,214630394	0,25557162
AMYLOID-BETA BINDING	15	1,764705882	3,365584109	3,13143E-05
GTPASE ACTIVATOR ACTIVITY	14	1,647058824	0,983979483	0,565129956
NEUROTRANSMITTER RECEPTOR ACTIVITY	14	1,647058824	3,17966593	0,000108687
SMALL GTPASE BINDING	14	1,647058824	1,091018798	0,408724482
IRON ION BINDING	14	1,647058824	2,085948347	0,007219503
PROTEIN-MACROMOLECULE ADAPTOR ACTIVITY	14	1,647058824	2,929607942	0,000266307
CARBOHYDRATE BINDING	14	1,647058824	1,473161985	0,095267815
ATPASE BINDING	13	1,529411765	2,848508045	0,000576716
TRANSMEMBRANE RECEPTOR PROTEIN TYROSINE KINASE ACTIVITY	13	1,529411765	2,105483601	0,00874615
STRUCTURAL MOLECULE ACTIVITY	13	1,529411765	1,32316086	0,186037337
MAGNESIUM ION BINDING	12	1,411764706	1,03966762	0,488428552
PHOSPHOLIPID BINDING	12	1,411764706	2,050627786	0,014016863

SEQUENCE-SPECIFIC DNA BINDING	12	1,411764706	0,916105631	0,665287731
EXTRACELLULAR MATRIX STRUCTURAL CONSTITUENT	12	1,411764706	2,213037669	0,007854907
VOLTAGE-GATED CALCIUM CHANNEL ACTIVITY	12	1,411764706	5,881055905	3,96428E-07
VIRUS RECEPTOR ACTIVITY	11	1,294117647	2,696042639	0,002355548
HYDROLASE ACTIVITY	11	1,294117647	2,561257355	0,003549204
TRANSCRIPTION CORE-PRESSOR ACTIVITY	11	1,294117647	1,113668828	0,402089529
HEME BINDING	11	1,294117647	1,463654032	0,132690501
SERINE-TYPE ENDOPEPTI-DASE ACTIVITY	11	1,294117647	1,249473819	0,267207748
HORMONE ACTIVITY	11	1,294117647	2,049057104	0,018302037
DNA-BINDING TRANSCRIP-TION REPRESSOR ACTIVITY, RNA POLYMERASE II-SPECIFIC	11	1,294117647	0,665323207	0,94600891
BETA-CATENIN BINDING	11	1,294117647	2,410612881	0,0056737
SERINE-TYPE ENDOPEPTI-DASE INHIBITOR ACTIVITY	10	1,176470588	1,862948375	0,042199296
PROTEIN TYROSINE KINASE ACTIVITY	10	1,176470588	2,517409364	0,006024298
MICROTUBULE BINDING	10	1,176470588	0,736387759	0,878690228
COLLAGEN BINDING	10	1,176470588	3,053818505	0,001414136
STRUCTURAL CONSTITUENT OF CYTOSKELETON	10	1,176470588	1,844505168	0,044685052
COPPER ION BINDING	10	1,176470588	3,449610572	0,000526754
CYSTEINE-TYPE ENDOPEPTI-DASE ACTIVITY	10	1,176470588	2,02492628	0,025642175
PROTEIN N-TERMINUS BIND-ING	9	1,058823529	1,612352015	0,106594737
CALCIUM-DEPENDENT PRO-TEIN BINDING	9	1,058823529	1,927373096	0,043632368
HISTONE BINDING	9	1,058823529	1,004135878	0,544908784
ATPASE-COUPLED TRANS-MEMBRANE TRANSPORTER ACTIVITY	9	1,058823529	3,644875745	0,000653609
CHOLESTEROL BINDING	9	1,058823529	3,287605039	0,001419738
LIPID TRANSPORTER ACTIV-ITY	9	1,058823529	6,705347183	3,34842E-06
ACETYLCHOLINE BINDING	9	1,058823529	13,9634249	6,80363E-10
ACETYLCHOLINE-GATED CATION-SELECTIVE CHAN-NEL ACTIVITY	9	1,058823529	10,4747491	2,90879E-08
EPIDERMAL GROWTH FAC-TOR RECEPTOR BINDING	9	1,058823529	5,08030091	4,28584E-05
ALDEHYDE DEHYDROGEN-ASE (NAD+) ACTIVITY	9	1,058823529	12,8901409	2,10548E-09

PROTON-TRANSPORTING ATP SYNTHASE ACTIVITY, ROTATIONAL MECHANISM	9	1,058823529	13,9634249	6,80363E-10
CYCLIN-DEPENDENT PROTEIN SERINE/THREONINE KINASE ACTIVITY	9	1,058823529	5,780790522	1,35131E-05

Supplementary Table-5: FunRich Analysis of Over-represented Proteins of the Biological Process in MSCs following estradiol treatment

---

BIOLOGICAL PROCESS	NO. OF PROTEINS IN THE DATASET	PERCENTAGE OF PROTEINS	FOLD ENRICHMENT	P-VALUE
SIGNAL TRANSDUCTION	92	10,57471264	1,874862069	3,18916E-09
G PROTEIN-COUPLED RECEPTOR SIGNALING PATHWAY	82	9,425287356	2,159146954	2,95236E-11
POSITIVE REGULATION OF TRANSCRIPTION BY RNA POLYMERASE II	79	9,08045977	1,564338632	4,70269E-05
NEUTROPHIL DEGRANULATION	67	7,701149425	2,719528525	6,50471E-14
REGULATION OF TRANSCRIPTION BY RNA POLYMERASE II	66	7,586206897	0,839031643	0,947829279
NEGATIVE REGULATION OF APOPTOTIC PROCESS	62	7,126436782	2,495896517	2,48914E-11
POSITIVE REGULATION OF CELL POPULATION PROLIFERATION	58	6,666666667	2,301744881	2,55414E-09
CELL ADHESION	54	6,206896552	2,169433526	6,91096E-08
APOPTOTIC PROCESS	53	6,091954023	1,949162546	2,58171E-06
RESPONSE TO DRUG	48	5,517241379	3,65419512	3,85807E-15
NEGATIVE REGULATION OF TRANSCRIPTION BY RNA POLYMERASE II	46	5,287356322	1,123632635	0,224145819
ION TRANSMEMBRANE TRANSPORT	45	5,172413793	4,974177284	1,06726E-19
POSITIVE REGULATION OF GENE EXPRESSION	43	4,942528736	2,151749085	1,8773E-06
POSITIVE REGULATION OF TRANSCRIPTION, DNA-TEMPLATED	42	4,827586207	1,505088834	0,005496002
POSITIVE REGULATION OF APOPTOTIC PROCESS	42	4,827586207	2,409881101	1,17546E-07
CELL DIFFERENTIATION	42	4,827586207	1,367354211	0,024865332
INFLAMMATORY RESPONSE	41	4,712643678	2,072896562	8,17379E-06
VIRAL PROCESS	38	4,367816092	1,581979387	0,003589858

PROTEIN PHOSPHORYLATION	38	4,367816092	2,009529828	3,47001E-05
INNATE IMMUNE RESPONSE	36	4,137931034	1,262383148	0,08957068
CELL SURFACE RECEPTOR SIGNALING PATHWAY	36	4,137931034	2,489037209	4,28016E-07
POSITIVE REGULATION OF CYTOSOLIC CALCIUM ION CONCENTRATION	33	3,793103448	4,333513548	5,90563E-13
RESPONSE TO HYPOXIA	33	3,793103448	4,112711634	2,76763E-12
IMMUNE RESPONSE	33	3,793103448	1,60228494	0,005281699
CELL-CELL SIGNALING	32	3,67816092	2,734269238	2,24174E-07
CYTOKINE-MEDIATED SIGNALING PATHWAY	30	3,448275862	1,983214374	0,000282828
NEGATIVE REGULATION OF CELL POPULATION PROLIFERATION	30	3,448275862	1,505221115	0,017109296
REGULATION OF TRANSCRIPTION, DNA-TEMPLATED	30	3,448275862	1,233275114	0,138537003
CHEMICAL SYNAPTIC TRANSMISSION	29	3,333333333	2,149499759	8,86663E-05
CELLULAR PROTEIN METABOLIC PROCESS	28	3,218390805	3,701963216	1,59999E-09
ANGIOGENESIS	28	3,218390805	2,392592357	1,70654E-05
ADAPTIVE IMMUNE RESPONSE	26	2,988505747	1,259384333	0,134963921
CELL DIVISION	26	2,988505747	1,445424461	0,038588093
POSITIVE REGULATION OF ERK1 AND ERK2 CASCADE	26	2,988505747	2,518706324	1,36512E-05
SPERMATOGENESIS	26	2,988505747	1,247037731	0,145792857
REGULATION OF APOPTOTIC PROCESS	26	2,988505747	2,388638395	3,47536E-05
MULTICELLULAR ORGANISM DEVELOPMENT	26	2,988505747	1,05340234	0,421391185
NEGATIVE REGULATION OF TRANSCRIPTION, DNA-TEMPLATED	25	2,873563218	0,953669536	0,627581822
NERVOUS SYSTEM DEVELOPMENT	25	2,873563218	1,443149196	0,0425438
NEGATIVE REGULATION OF INFLAMMATORY RESPONSE TO ANTIGENIC STIMULUS	25	2,873563218	2,374845924	5,37881E-05
POST-TRANSLATIONAL PROTEIN MODIFICATION	25	2,873563218	1,4221738	0,049064696
INTRACELLULAR PROTEIN TRANSPORT	24	2,75862069	1,683381073	0,009001882
NEGATIVE REGULATION OF GENE EXPRESSION	24	2,75862069	2,257969103	0,000166042
CELLULAR RESPONSE TO DNA DAMAGE STIMULUS	24	2,75862069	1,886189925	0,002180068

POSITIVE REGULATION OF CELL MIGRATION	24	2,75862069	2,033159401	0,000781113
EXTRACELLULAR MATRIX ORGANIZATION	23	2,643678161	1,793228547	0,004981912
POSITIVE REGULATION OF PROTEIN KINASE B SIGNAL-ING	23	2,643678161	2,542897563	3,62291E-05
MEMBRANE ORGANIZATION	23	2,643678161	3,629693554	6,69639E-08
CELL MIGRATION	22	2,528735632	1,855766743	0,00396844
PLATELET DEGRANULATION	22	2,528735632	3,444176002	3,35396E-07
ADENYLATE CYCLASE-ACTI-VATING G PROTEIN-COU-PLED RECEPTOR SIGNALING PATHWAY	22	2,528735632	3,648474214	1,17102E-07
REGULATION OF IMMUNE RESPONSE	22	2,528735632	2,059980106	0,001069158
RESPONSE TO ETHANOL	21	2,413793103	3,989851339	4,5194E-08
AGING	21	2,413793103	2,617633185	5,08766E-05
POSITIVE REGULATION OF ANGIOGENESIS	21	2,413793103	3,13712378	2,97569E-06
POSITIVE REGULATION OF PROTEIN PHOSPHORYLA-TION	21	2,413793103	2,270562877	0,000385165
REGULATION OF GENE EX-PRESSION	21	2,413793103	1,726795456	0,010669353
LEUKOCYTE MIGRATION	20	2,298850575	2,017590489	0,002262449
PROTEOLYSIS	20	2,298850575	1,547103793	0,035652463
MAPK CASCADE	19	2,183908046	1,367122073	0,104557025
INTRACELLULAR SIGNAL TRANSDUCTION	19	2,183908046	0,991629224	0,549851836
IN UTERO EMBRYONIC DE-VELOPMENT	19	2,183908046	2,124855009	0,001588564
HEART DEVELOPMENT	19	2,183908046	1,946866003	0,004267942
RESPONSE TO LIPOPOLYSAC-CHARIDE	19	2,183908046	2,694521232	7,7039E-05
REGULATION OF COMPLE-MENT ACTIVATION	19	2,183908046	3,319979244	3,70665E-06
PROTEIN STABILIZATION	18	2,068965517	1,797403293	0,011805947
PROTEIN AUTOPHOSPHORY-LATION	18	2,068965517	2,036350613	0,003315938
SKELETAL SYSTEM DEVELOP-MENT	18	2,068965517	2,773868353	8,05671E-05
ACTIN FILAMENT ORGANI-ZATION	17	1,954022989	2,619850122	0,00025473
CALCIUM ION TRANSPORT	17	1,954022989	4,377676147	2,1078E-07
REGULATION OF SMALL GTPASE MEDIATED SIGNAL TRANSDUCTION	17	1,954022989	2,428634143	0,000624811
CELLULAR RESPONSE TO LIP-OPOLYSACCHARIDE	17	1,954022989	2,174675929	0,002137849

BMP SIGNALING PATHWAY	17	1,954022989	4,557556005	1,12634E-07
PROTEIN-CONTAINING COMPLEX ASSEMBLY	16	1,83908046	2,485400431	0,000692574
CELLULAR CALCIUM ION HOMEOSTASIS	16	1,83908046	3,228381696	3,06194E-05
POSITIVE REGULATION OF PEPTIDYL-TYROSINE PHOS-PHORYLATION	16	1,83908046	3,641266229	6,3173E-06
LOCOMOTORY BEHAVIOR	16	1,83908046	4,538259793	2,82646E-07
DNA REPAIR	16	1,83908046	1,423504879	0,099185833
BLOOD COAGULATION	16	1,83908046	1,831385933	0,014412001
POSITIVE REGULATION OF NF-KAPPAB TRANSCRIPTION FACTOR ACTIVITY	16	1,83908046	1,933123315	0,008872985
CELLULAR RESPONSE TO ME-CHANICAL STIMULUS	16	1,83908046	4,23166205	7,81517E-07
LIPID METABOLIC PROCESS	16	1,83908046	2,302663836	0,001579132
VISUAL PERCEPTION	16	1,83908046	1,527658691	0,061182579
REGULATION OF CARDIAC CONDUCTION	16	1,83908046	5,908042036	4,73609E-09
ACTIN CYTOSKELETON OR-GANIZATION	16	1,83908046	2,115973977	0,003744281
PROTEIN DEUBIQUITINA-TION	16	1,83908046	1,181786756	0,281787761
CELL-CELL ADHESION	16	1,83908046	2,39054506	0,001059481
RECEPTOR-MEDIATED EN-DOCYTOSIS	16	1,83908046	1,957285847	0,007910805
CELL POPULATION PROLIF-ERATION	16	1,83908046	2,372436242	0,001149827
PROTON TRANSMEMBRANE TRANSPORT	16	1,83908046	6,390232776	1,29533E-09
REGULATION OF CELL CYCLE	16	1,83908046	2,954299673	9,26446E-05
REGULATION OF CYTOSOLIC CALCIUM ION CONCENTRA-TION	15	1,724137931	7,159801334	6,86833E-10
CELLULAR RESPONSE TO HY-POXIA	15	1,724137931	2,575418408	0,000692245
POSITIVE REGULATION OF I-KAPPAB KINASE/NF-KAPPAB SIGNALING	15	1,724137931	1,604412069	0,048486392
REGULATION OF MEM-BRANE POTENTIAL	15	1,724137931	2,995851982	0,000129563
RESPONSE TO ESTRADIOL	15	1,724137931	3,226276813	5,42414E-05
PROTEIN TRANSPORT	15	1,724137931	0,889741077	0,717081226
POSITIVE REGULATION OF MAPK CASCADE	15	1,724137931	2,645018041	0,000520402
POSITIVE REGULATION OF PEPTIDYL-SERINE PHOS-PHORYLATION	15	1,724137931	3,26212035	4,7509E-05

NEGATIVE REGULATION OF NEURON APOPTOTIC PROCESS	15	1,724137931	2,311813658	0,002098101
XENOBIOTIC METABOLIC PROCESS	15	1,724137931	3,336251025	3,62073E-05
BRAIN DEVELOPMENT	15	1,724137931	1,328552793	0,161415291
REGULATION OF INFLAMMATORY RESPONSE	15	1,724137931	3,537205791	1,76177E-05
TRANSMEMBRANE RECEPTOR PROTEIN TYROSINE KINASE SIGNALING PATHWAY	15	1,724137931	2,488123487	0,000994521
TRANSCRIPTION BY RNA POLYMERASE II	15	1,724137931	1,649477292	0,039613513
REGULATION OF BLOOD PRESSURE	15	1,724137931	4,317357046	1,31529E-06
SENSORY PERCEPTION OF SOUND	15	1,724137931	2,11224698	0,004994271
ANTIGEN PROCESSING AND PRESENTATION OF EXOGENOUS PEPTIDE ANTIGEN VIA MHC CLASS II	15	1,724137931	2,965593907	0,000145616
TRANSFORMING GROWTH FACTOR BETA RECEPTOR SIGNALING PATHWAY	15	1,724137931	2,965593907	0,000145616
OSTEOBLAST DIFFERENTIATION	15	1,724137931	2,850436392	0,00022834
PEPTIDYL-SERINE PHOSPHORYLATION	15	1,724137931	1,758118991	0,024290685
HOMOPHILIC CELL ADHESION VIA PLASMA MEMBRANE ADHESION MOLECULES	15	1,724137931	1,768709431	0,023158794
INSULIN RECEPTOR SIGNALING PATHWAY	14	1,609195402	3,425341796	4,84079E-05
COLLAGEN FIBRIL ORGANIZATION	14	1,609195402	2,768019363	0,000496497
POSITIVE REGULATION OF NEURON DIFFERENTIATION	14	1,609195402	3,149771257	0,000124594
APOPTOTIC SIGNALING PATHWAY	14	1,609195402	4,349493685	2,69288E-06
ACTIVATION OF ADENYLATE CYCLASE ACTIVITY	14	1,609195402	6,682799247	7,26005E-09
RESPONSE TO GLUCOCORTICOID	14	1,609195402	5,372703335	1,65068E-07
FEMALE PREGNANCY	14	1,609195402	3,383058846	5,5812E-05
ACTIVATION OF CYSTEINE-TYPE ENDOPEPTIDASE ACTIVITY INVOLVED IN APOPTOTIC PROCESS	14	1,609195402	3,078997833	0,000159944
VESICLE-MEDIATED TRANSPORT	14	1,609195402	1,779505208	0,026382743

RHO PROTEIN SIGNAL TRANSDUCTION	14	1,609195402	5,169998059	2,78998E-07
COMPLEMENT ACTIVATION, CLASSICAL PATHWAY	14	1,609195402	1,889949639	0,016556266
REGULATION OF CYCLIN-DEPENDENT PROTEIN SERINE/THREONINE KINASE ACTIVITY	13	1,494252874	4,989212733	1,16051E-06
PLATELET ACTIVATION	13	1,494252874	2,378279988	0,003158521
REGULATION OF SIGNAL TRANSDUCTION BY P53 CLASS MEDIATOR	13	1,494252874	1,663288292	0,050023679
G1/S TRANSITION OF MITOTIC CELL CYCLE	13	1,494252874	3,687867577	4,01353E-05
POSITIVE REGULATION OF VASOCONSTRICTION	13	1,494252874	8,480497884	7,93507E-10
ACTIVATION OF MAPK ACTIVITY	13	1,494252874	2,156594708	0,007272479
ENDOCYTOSIS	13	1,494252874	1,719476667	0,040062449
NOTCH SIGNALING PATHWAY	13	1,494252874	2,494850912	0,002059451
LIVER DEVELOPMENT	13	1,494252874	3,438721004	8,582E-05
RESPONSE TO NUTRIENT	13	1,494252874	3,975937221	1,72808E-05
CELL CYCLE ARREST	13	1,494252874	1,957539739	0,015696236
CHOLESTEROL HOMEOSTASIS	13	1,494252874	3,065892561	0,000283845
REGULATION OF METABOLIC PROCESS	13	1,494252874	4,387170169	5,51566E-06
RESPONSE TO CALCIUM ION	13	1,494252874	4,626426859	2,92444E-06
INTEGRIN-MEDIATED SIGNALING PATHWAY	13	1,494252874	2,736262138	0,000870861
EPITHELIAL CELL DIFFERENTIATION	13	1,494252874	3,797936748	2,89589E-05
PROTEIN LOCALIZATION	13	1,494252874	1,885043637	0,020851047
ADHERENS JUNCTION ORGANIZATION	13	1,494252874	5,917222541	1,30389E-07
RENAL WATER HOMEOSTASIS	13	1,494252874	7,067474077	1,14987E-08
RESPONSE TO TOXIC SUBSTANCE	13	1,494252874	3,348240251	0,000113962
REGULATION OF CELL SHAPE	13	1,494252874	1,755049593	0,03480767
INTRINSIC APOPTOTIC SIGNALING PATHWAY IN RESPONSE TO DNA DAMAGE	12	1,379310345	5,592427657	7,87623E-07
POSITIVE REGULATION OF NEURON PROJECTION DEVELOPMENT	12	1,379310345	2,042760507	0,014557408
NEGATIVE REGULATION OF CELL GROWTH	12	1,379310345	1,909908836	0,023538696

ENDOPLASMIC RETICULUM TO GOLGI VESICLE-MEDIATED TRANSPORT	12	1,379310345	1,276766947	0,232633773
CELLULAR RESPONSE TO CAMP	12	1,379310345	4,270821412	1,69156E-05
NEGATIVE REGULATION OF CELL MIGRATION	12	1,379310345	2,02515202	0,015508823
ATP BIOSYNTHETIC PROCESS	12	1,379310345	8,098513819	6,845E-09
CELLULAR RESPONSE TO CALCIUM ION	12	1,379310345	2,830235946	0,001005135
WNT SIGNALING PATHWAY	12	1,379310345	1,249603138	0,25497756
TRANSMEMBRANE TRANSPORT	12	1,379310345	1,459150897	0,122483025
RETINOID METABOLIC PROCESS	12	1,379310345	3,728580954	7,06141E-05
PROTEIN LOCALIZATION TO PLASMA MEMBRANE	12	1,379310345	1,566148163	0,083016837
POTASSIUM ION IMPORT ACROSS PLASMA MEMBRANE	12	1,379310345	5,592427657	7,87623E-07
T CELL RECEPTOR SIGNALING PATHWAY	12	1,379310345	1,319801617	0,200681404
RECEPTOR INTERNALIZATION	12	1,379310345	5,219681979	1,77753E-06
POSITIVE REGULATION OF PATHWAY-RESTRICTED SMAD PROTEIN PHOSPHORYLATION	12	1,379310345	4,893519806	3,73693E-06
POSITIVE REGULATION OF OSTEOBLAST DIFFERENTIATION	12	1,379310345	3,981323265	3,57541E-05
EXTRINSIC APOPTOTIC SIGNALING PATHWAY IN ABSENCE OF LIGAND	12	1,379310345	7,828653312	1,08793E-08
COMPLEMENT ACTIVATION	12	1,379310345	2,472770086	0,003246964
CELLULAR RESPONSE TO INSULIN STIMULUS	11	1,264367816	2,220142565	0,010548239
ADENYLATE CYCLASE-INHIBITING G PROTEIN-COUPLED RECEPTOR SIGNALING PATHWAY	11	1,264367816	3,473246739	0,00027114
PHOSPHOLIPASE C-ACTIVATING G PROTEIN-COUPLED RECEPTOR SIGNALING PATHWAY	11	1,264367816	3,530175877	0,000233664
POSITIVE REGULATION OF ENDOTHELIAL CELL PROLIFERATION	11	1,264367816	3,076360952	0,000795947
NERVOUS SYSTEM PROCESS	11	1,264367816	4,222231529	4,24464E-05
DEFENSE RESPONSE TO VIRUS	11	1,264367816	1,071469232	0,452373725

MALE GONAD DEVELOPMENT	11	1,264367816	2,266877489	0,009073952
CHOLESTEROL METABOLIC PROCESS	11	1,264367816	2,796728091	0,00177738
DEFENSE RESPONSE TO GRAM-POSITIVE BACTERIUM	11	1,264367816	2,132224832	0,01404055
POSITIVE REGULATION OF NEURON DEATH	11	1,264367816	5,251792984	4,49887E-06
CENTRAL NERVOUS SYSTEM DEVELOPMENT	11	1,264367816	1,57197307	0,09230113
EPIDERMAL GROWTH FACTOR RECEPTOR SIGNALING PATHWAY	11	1,264367816	4,394532346	2,85142E-05
GLUCOSE HOMEOSTASIS	11	1,264367816	2,07072426	0,017185419
REGULATION OF CELL POPULATION PROLIFERATION	11	1,264367816	1,549356379	0,099651553
BLOOD VESSEL REMODELING	11	1,264367816	6,332726559	5,66112E-07
RESPONSE TO VIRUS	11	1,264367816	1,975745622	0,023552892
CELLULAR RESPONSE TO BMP STIMULUS	11	1,264367816	6,52456923	4,01415E-07
POSITIVE REGULATION OF BONE MINERALIZATION	11	1,264367816	5,666299139	1,97274E-06
ANIMAL ORGAN REGENERATION	11	1,264367816	5,126780059	5,81576E-06
NEGATIVE REGULATION OF ANGIOGENESIS	11	1,264367816	2,419683522	0,00558807
CELLULAR SODIUM ION HOMEOSTASIS	11	1,264367816	11,32961758	3,03508E-10
OSSIFICATION	11	1,264367816	2,796728091	0,00177738
REGULATION OF DEFENSE RESPONSE TO VIRUS BY VIRUS	11	1,264367816	7,9739367	3,58792E-08
WOUND HEALING	11	1,264367816	2,340789374	0,007166911
CEREBRAL CORTEX DEVELOPMENT	11	1,264367816	3,364724735	0,000361426
SODIUM ION EXPORT ACROSS PLASMA MEMBRANE	11	1,264367816	15,37302143	1,85197E-12
CELLULAR RESPONSE TO AMINO ACID STARVATION	11	1,264367816	2,691863895	0,002426495
TRANSFERRIN TRANSPORT	11	1,264367816	5,981006117	1,08207E-06
NEGATIVE REGULATION OF CYSTEINE-TYPE ENDOPEPTIDASE ACTIVITY INVOLVED IN APOPTOTIC PROCESS	10	1,149425287	3,625517028	0,000356421
CELLULAR RESPONSE TO GROWTH FACTOR STIMULUS	10	1,149425287	3,559610519	0,000415967
MITOTIC CELL CYCLE	10	1,149425287	1,472176338	0,143301407

REGULATION OF ACTIN CYTOSKELETON ORGANIZATION	10	1,149425287	2,682018555	0,003865025
CELLULAR RESPONSE TO AMYLOID-BETA	10	1,149425287	5,593092678	6,60253E-06
NEURON APOPTOTIC PROCESS	10	1,149425287	4,894130834	2,41558E-05
POSITIVE REGULATION OF NEURON APOPTOTIC PROCESS	10	1,149425287	3,99539226	0,000154236
POSITIVE REGULATION OF PROTEIN SECRETION	10	1,149425287	4,165372786	0,000106619
TRANSPORT ACROSS BLOOD-BRAIN BARRIER	10	1,149425287	2,330843646	0,010406573
FATTY ACID METABOLIC PROCESS	10	1,149425287	2,645779958	0,004269344
REGULATION OF MACROAUTOPHAGY	10	1,149425287	2,719263639	0,003491602
CELLULAR RESPONSE TO HYDROGEN PEROXIDE	10	1,149425287	3,15778382	0,001107531
LIPID TRANSPORT	10	1,149425287	2,837475361	0,002540058
REGULATION OF INSULIN SECRETION	10	1,149425287	2,879196804	0,002273778
RESPONSE TO ORGANIC CYCLIC COMPOUND	10	1,149425287	4,449310944	5,87174E-05
ANIMAL ORGAN MORPHOGENESIS	10	1,149425287	1,450367933	0,153289365
KIDNEY DEVELOPMENT	10	1,149425287	1,864719309	0,042211213
T CELL ACTIVATION	10	1,149425287	4,552759234	4,75286E-05
ODONTOGENESIS OF DENTIN-CONTAINING TOOTH	10	1,149425287	3,764933179	0,000258436
CRISTAE FORMATION	10	1,149425287	6,314549328	1,92234E-06
CELL-CELL ADHESION VIA PLASMA-MEMBRANE ADHESION MOLECULES	10	1,149425287	5,290845033	1,14134E-05
CELLULAR RESPONSE TO TUMOR NECROSIS FACTOR	10	1,149425287	1,553957421	0,111146395
CALCIUM ION TRANSMEMBRANE TRANSPORT	10	1,149425287	3,15778382	0,001107531
CELLULAR POTASSIUM ION HOMEOSTASIS	10	1,149425287	15,05105109	2,88095E-11
ATP METABOLIC PROCESS	10	1,149425287	6,524964167	1,36447E-06
MITOCHONDRIAL ATP SYNTHESIS COUPLED PROTON TRANSPORT	10	1,149425287	10,30058783	7,02052E-09
CARTILAGE DEVELOPMENT	10	1,149425287	3,375524473	0,000646181
REGULATION OF HEART RATE BY CARDIAC CONDUCTION	9	1,034482759	4,518131737	0,000121065

POSITIVE REGULATION OF DNA-BINDING TRANSCRIPTION FACTOR ACTIVITY	9	1,034482759	1,573540925	0,119408007
ACTIVATION OF PHOSPHOLIPASE C ACTIVITY	9	1,034482759	5,506164294	2,21167E-05
CIRCADIAN RHYTHM	9	1,034482759	2,554011289	0,008262604
POSITIVE REGULATION OF SMOOTH MUSCLE CELL PROLIFERATION	9	1,034482759	3,263327514	0,00152972
AMYLOID FIBRIL FORMATION	9	1,034482759	2,753512249	0,005032156
ANTIBACTERIAL HUMORAL RESPONSE	9	1,034482759	3,388815979	0,001159261
ANTIMICROBIAL HUMORAL IMMUNE RESPONSE MEDIATED BY ANTIMICROBIAL PEPTIDE	9	1,034482759	1,711021445	0,080732699
DEFENSE RESPONSE TO GRAM-NEGATIVE BACTERIUM	9	1,034482759	2,04920729	0,031320746
NEGATIVE REGULATION OF CANONICAL WNT SIGNALING PATHWAY	9	1,034482759	0,995719559	0,554988993
POSITIVE REGULATION OF PROTEIN BINDING	9	1,034482759	2,554011289	0,008262604
POST-GOLGI VESICLE-MEDIATED TRANSPORT	9	1,034482759	3,038309241	0,002553752
PHAGOCYTOSIS	9	1,034482759	2,937049143	0,003237601
ADENYLATE CYCLASE-MODULATING G PROTEIN-COUPLED RECEPTOR SIGNALING PATHWAY	9	1,034482759	3,388815979	0,001159261
SYNAPTIC TRANSMISSION, CHOLINERGIC	9	1,034482759	9,271558078	1,32935E-07
POSITIVE REGULATION OF ENDOTHELIAL CELL MIGRATION	9	1,034482759	2,842320901	0,004057894
CELLULAR RESPONSE TO OXIDATIVE STRESS	9	1,034482759	1,798309551	0,063041341
INTRACELLULAR RECEPTOR SIGNALING PATHWAY	9	1,034482759	3,388815979	0,001159261
FATTY ACID BETA-OXIDATION	9	1,034482759	3,915848013	0,000384462
PEPTIDYL-TYROSINE PHOSPHORYLATION	9	1,034482759	3,146800912	0,001989684
CYTOSKELETON ORGANIZATION	9	1,034482759	1,587715693	0,11468314
B CELL RECEPTOR SIGNALING PATHWAY	9	1,034482759	1,573540925	0,119408007
PROTEIN HOMOTETRAMERIZATION	9	1,034482759	3,146800912	0,001989684

SMAD PROTEIN SIGNAL TRANSDUCTION	9	1,034482759	3,038309241	0,002553752
POSITIVE REGULATION OF B CELL PROLIFERATION	9	1,034482759	4,297788809	0,000182585
MUSCLE CONTRACTION	9	1,034482759	1,694570898	0,084597462
GENERATION OF PRECURSOR METABOLITES AND ENERGY	9	1,034482759	3,749251628	0,000539483
ETHANOL OXIDATION	9	1,034482759	14,6754637	4,38387E-10
NEURON DIFFERENTIATION	9	1,034482759	1,198913809	0,337894538
TUMOR NECROSIS FACTOR-MEDIATED SIGNALING PATHWAY	9	1,034482759	1,468646938	0,1608601
PEPTIDYL-THREONINE PHOSPHORYLATION	9	1,034482759	2,630239055	0,006822337
NEGATIVE REGULATION OF CELL ADHESION	9	1,034482759	4,097938132	0,000268134
CELL-MATRIX ADHESION	9	1,034482759	1,894982465	0,048034401
CELL MORPHOGENESIS	9	1,034482759	2,349717625	0,013981632
MULTICELLULAR ORGANISM GROWTH	9	1,034482759	2,414084633	0,011822736
HEMOPOIESIS	9	1,034482759	3,388815979	0,001159261
REGULATION OF TRANSCRIPTION INITIATION FROM RNA POLYMERASE II PROMOTER	9	1,034482759	3,263327514	0,00152972
POSITIVE REGULATION OF CANONICAL WNT SIGNALING PATHWAY	9	1,034482759	1,198913809	0,337894538
CALCIUM-DEPENDENT CELL-CELL ADHESION VIA PLASMA MEMBRANE CELL ADHESION MOLECULES	9	1,034482759	4,195484862	0,000221961
T CELL COSTIMULATION	9	1,034482759	3,455250325	0,001003349
TRANSCRIPTION INITIATION FROM RNA POLYMERASE II PROMOTER	9	1,034482759	1,258855218	0,288038364
POSITIVE REGULATION OF FIBROBLAST PROLIFERATION	9	1,034482759	4,004824337	0,000321984
WNT SIGNALING PATHWAY, PLANAR CELL POLARITY PATHWAY	9	1,034482759	2,025655891	0,033416058
RESPONSE TO ESTROGEN	9	1,034482759	2,986821201	0,002879684
WATER TRANSPORT	9	1,034482759	11,00888938	1,89246E-08
CELLULAR RESPONSE TO UV	9	1,034482759	3,324888116	0,00133417
POSITIVE REGULATION OF EPITHELIAL CELL PROLIFERATION	9	1,034482759	3,091603562	0,002257749
COMPLEMENT ACTIVATION, ALTERNATIVE PATHWAY	9	1,034482759	11,74232639	8,67359E-09
